# Progress and prospects of mRNA-based drugs in pre-clinical and clinical applications

**DOI:** 10.1038/s41392-024-02002-z

**Published:** 2024-11-14

**Authors:** Yingying Shi, Meixing Shi, Yi Wang, Jian You

**Affiliations:** 1 College of Pharmaceutical Sciences, Zhejiang University, 866 Yuhangtang Road, Hangzhou, Zhejiang P. R. China; 2 State Key Laboratory for Diagnosis and Treatment of Infectious Diseases, 79 Qingchun Road, Shangcheng District, Hangzhou, Zhejiang P. R. China; 3 The First Affiliated Hospital, College of Medicine, Zhejiang University, 79 QingChun Road, Hangzhou, Zhejiang P. R. China; 4 Jinhua Institute of Zhejiang University, 498 Yiwu Street, Jinhua, Zhejiang P. R. China

**Keywords:** Nucleic-acid therapeutics, Preclinical research, Translational research, Genetics research

## Abstract

In the last decade, messenger ribonucleic acid (mRNA)-based drugs have gained great interest in both immunotherapy and non-immunogenic applications. This surge in interest can be largely attributed to the demonstration of distinct advantages offered by various mRNA molecules, alongside the rapid advancements in nucleic acid delivery systems. It is noteworthy that the immunogenicity of mRNA drugs presents a double-edged sword. In the context of immunotherapy, extra supplementation of adjuvant is generally required for induction of robust immune responses. Conversely, in non-immunotherapeutic scenarios, immune activation is unwanted considering the host tolerability and high expression demand for mRNA-encoded functional proteins. Herein, mainly focused on the linear non-replicating mRNA, we overview the preclinical and clinical progress and prospects of mRNA medicines encompassing vaccines and other therapeutics. We also highlight the importance of focusing on the host-specific variations, including age, gender, pathological condition, and concurrent medication of individual patient, for maximized efficacy and safety upon mRNA administration. Furthermore, we deliberate on the potential challenges that mRNA drugs may encounter in the realm of disease treatment, the current endeavors of improvement, as well as the application prospects for future advancements. Overall, this review aims to present a comprehensive understanding of mRNA-based therapies while illuminating the prospective development and clinical application of mRNA drugs.

## Introduction

Recently, messenger ribonucleic acid (mRNA) therapy represents a novel approach for treating a wide range of diseases, encompassing both immune-related and non-immune conditions. Amidst the COVID-19 pandemic, mRNA vaccines have achieved remarkable advancements, owing to the unwavering dedication of numerous scientists who have been at the forefront of mRNA research for decades.

However, despite a plethora of preclinical studies conducted, successful translation of mRNA medicines into clinical applications remains limited, probably due to the suboptimal design and administration of mRNA drugs to patients with specific physiological and pathological conditions. Distinct characteristics of mRNA drugs are demanded in different therapeutic indications.^1^ Specifically, in immunogenic applications that include cancer immunotherapies and infectious disease vaccines, appropriate incorporation of adjuvants is required for eliciting augmented host immune responses. In contrast, in non-immunotherapies encompassing protein replacement/supplementation therapy, regenerative medicine therapy, and genetic editing, the immunogenicity of mRNA drugs is unfavorable, which may lead to diminished protein expression and even cause adverse reactions. On the other hand, the host individual variations, such as, age, gender, disease and medical history may influence both the efficacy and safety of mRNA medication. For example, compared to healthy adults, immunocompromised patients exhibit inadequate immune responses following initial mRNA vaccine inoculation, where repeated vaccinations are recommended for the establishment of sufficient immune protection.^2^ Nevertheless, repeated doses may cause potential risks that exacerbate the pathological burden.^3^ To date, there is still a lack of systemic understanding over the fate and outcomes of mRNA drugs in personalized recipient.

In this review, we first summarize the preclinical and clinical applications of mRNA drugs pertaining to immunotherapy and non-immunotherapy, then discuss the impact from patient physiological and pathological characteristics. Finally, we provide insights into the future directions and research priorities of mRNA drugs.

## Evolution and milestones in mRNA-based drugs

### Brief history of mRNA drug development

**Fig. 1:**
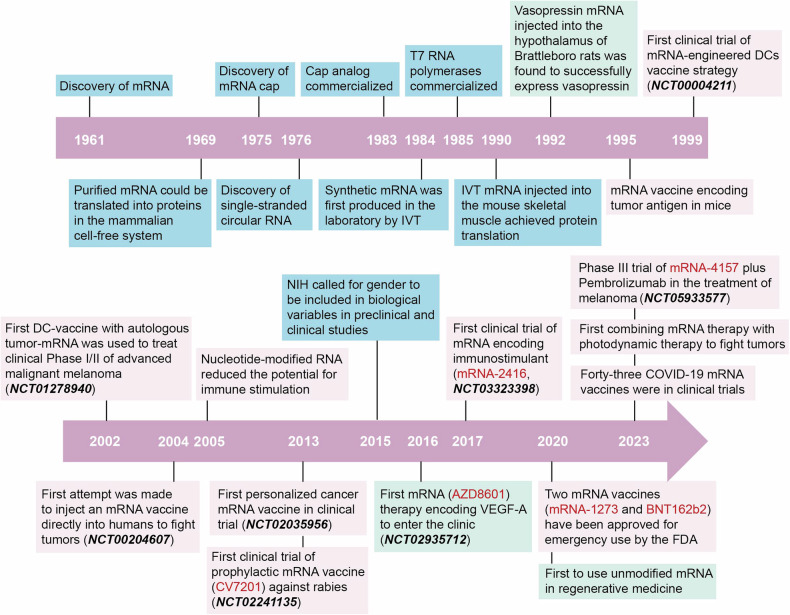
Chronological development of mRNA drugs. Yellow box, common events of mRNA drugs; green box, mRNA-based non-immunotherapy; red box, mRNA-based immunotherapy. From 1961 to 1990: mRNA discovery and the maturation of IVT mRNA technology, including the discovery of mRNA,^4^ purified mRNA could be translated into proteins in the mammalian cell-free system,^518^ discovery of mRNA cap,^519^ discovery of single-stranded circular RNA,^520^ cap analog commercialized,^18^ synthetic mRNA was first produced in the laboratory by IVT,^374^ T7 RNA polymerases commercialized.^335^ From 1990 to 2019: the exploration of mRNA vaccines, particularly for cancer therapy, including IVT mRNA injected into the mouse skeletal muscle achieved protein translation,^5^ vasopressin mRNA injected into the hypothalamus of Brattleboro rats was found to successfully express vasopressin,^357^ mRNA vaccine encoding tumor antigen in mice,^171^ first clinical trial of mRNA-engineered DCs vaccine strategy,^6–8^ first DC vaccine with autologous tumor mRNA was used to treat clinical Phase I/II of advanced malignant melanoma trial (NCT01278940),^332,333^ first attempt was made to inject an mRNA vaccine directly into humans to fight tumors (NCT00204607),^9,10^ nucleotide-modified RNA reduced the potential for immune stimulation,^11^ first personalized cancer mRNA vaccine in clinical trial (NCT02035956),^521,522^ first clinical trial of prophylactic mRNA vaccine (CV7201) against rabies (NCT02241135),^13^ NIH called for gender to be included in biological variables in preclinical and clinical studies,^435^ first mRNA (AZD8601) therapy encoding VEGF-A to enter the clinic (NCT02935712),^523^ first clinical trial of mRNA-encoding immunostimulant (mRNA-2416, NCT03323398).^524^ From 2019 to the present: rapid development of mRNA-based therapeutics, including two mRNA vaccines (mRNA-1273^486^ and BNT162b2) have been approved for emergency use by the FDA,^123,124^ first to use unmodified mRNA in regenerative medicine,^370^ forty-three COVID-19 mRNA vaccines were in clinical trials,^125^ first combining mRNA therapy with photodynamic therapy to fight tumors,^290^ Phase III trial of mRNA-4157 plus Pembrolizumab in the treatment of melanoma.^501^ IVT in vitro transcription, NIH the National Institutes of Health, DCs dendritic cells, FDA the Food and Drug Administration. The graphic is created with Adobe Illustrator

Chronologically (Fig. 1), mRNA was first discovered by pioneering researches in 1961.^4^ However, it was not until 1990 when Wolff et al. successfully expressed proteins by injecting mRNA into the body^5^ that mRNA gradually gained recognition as a therapeutic modality.^1^ In 1999, mRNA-engineered dendritic cells (DCs) entered clinical trial as antitumor vaccines for the first time (NCT00004211).^6–8^ In 2004, Weide et al. conducted the initial clinical trial involving the direct injection of protamine-stabilized mRNA into the human body to target metastatic melanoma (NCT00204607),^9^ wherein the mRNA encoded tumor-associated antigens (TAAs)—Melan-A, Mage-A1, Mage-A3, Tyrosinase, gp100, and Survivin.^10^ In 2005, Karikó et al. demonstrated that RNA could evade immune detection when it was naturally modified with nucleotides such as 5-methylcytosine (m^5^C), 5-methyluridine (m^5^U), *N*^6^-methyladenosine (m^6^A), pseudouridine (Ψ), and 2′-*O*-Methyluridine.^11,12^ The identification of this approach to mitigate the immunogenicity of mRNA is crucial for broadening the scope of mRNA therapeutics in non-immunological medical fields. In 2013, CureVac conducted an initial evaluation of the safety and immunogenicity of a prophylactic mRNA vaccine coding the rabies virus glycoprotein (CV7201) in a clinical trial (NCT02241135) involving healthy adults.^13^ This trial represents the first clinical study utilizing mRNA vaccines to combat infectious diseases.^12^ Then, in 2016, the therapeutic potential of mRNA was first unleashed in the field of protein supplementation therapy (NCT02935712). Notably, in 2020, two antivirus mRNA vaccines—mRNA-1273 by Moderna (NCT04283461) and BNT162b2 by Pfizer/BioNTech (NCT04380701), were quickly approved by the United States (U.S.) Food and Drug Administration (FDA) and put into use during the COVID-19 outbreak, which achieved great success and marked a significant milestone in emergency response against infectious diseases.^14^ Since then, mRNA drugs have experienced an explosion of development.

### Classification, advantages, and limitations of mRNA drugs

mRNA, a single-stranded ribonucleic acid, acts as a transient carrier for the genetic information transcribed from DNA to guide protein synthesis.^15^ The execution of mRNA function in eukaryotes involves multiple stages, including transcription, post-transcriptional processing, intracellular transport, and translation.^16^ Prior to the maturation of mRNA, precursor messenger RNA (pre-mRNA) must undergo three essential processing steps: addition of a 5’ cap structure, cleavage mediated by numerous protein factors, and 3’-end processing which includes adding a polyadenosine [poly(A)] tail. These pre-mRNA processing steps are crucial for the generation of mature mRNA.^17^ With the commercialization of Cap analogs and T7 RNA polymerase in 1983 and 1985, respectively, the technology for in vitro transcription (IVT) of mRNA has steadily matured.^18^ The structural characteristics of IVT mRNA closely resemble those of endogenous mRNA found in natural eukaryotic cells.^19^

The linear non-replicating mRNA, representative of IVT mRNA, is composed of five segments in the 5’ to 3’ direction: the 5’ cap, the 5’ untranslated region (UTR), an open reading frame (ORF) encoding the target protein, the 3’ UTR, and a poly(A) tail.^20–22^ The ORF functions as the coding sequence for protein translation and is an indispensable element of IVT mRNA. The UTR, although non-coding, plays a pivotal role in ribosome recruitment and successful mRNA translation. Furthermore, the 5’ cap and 3’ Poly(A) tail structures significantly contribute to enhancing mRNA stability and translation efficiency.^23,24^ One of the key attributes of mRNA is its highly customizable nature. The optimization of mRNA involves nucleotide sequence refinement, chemical modifications,^25^ and mRNA purification. Various factors must be taken into consideration during mRNA design, including the influence of nucleotide sequence on RNA folding, immunogenicity, enhancement of mRNA stability, and maximization of the expression of the target protein.^26,27^ The immunogenicity of exogenous mRNA elicits an innate immune response, leading to the suppression of exogenous mRNA translation in the body.^28^ Substituting uridine with modified nucleotides such as m^5^U and Ψ, which are not recognized by pattern-recognition receptors, can mitigate the immunogenicity of mRNA.^26^ In addition, several studies suggest that Ψ modification may enhance the stability of transcripts.^29^ Nevertheless, there have been investigations suggesting that specific circumstances may disrupt the stability of mRNA.^30^ It is noteworthy that there are currently more than 170 identified types of RNA modifications.^31^ Exploring the optimization of mRNA holds substantial research potential.^32^

Compared to recombinant protein drugs, mRNA drugs have a shorter production cycle^33^ and are not limited to short peptide sequences.^34^ mRNA vaccines can easily deliver numerous tumor antigen fragments simultaneously, thereby increasing the diversity of antigenic epitopes.^33^ In contrast to DNA vaccines, mRNA vaccines circumvent the need to enter the nucleus, thereby obviating the potential for gene insertion and subsequent mutations.^33,35^ In addition, mRNA exhibits modifiable immunogenicity^36^ and can be effectively potentiated by adjuvants to efficiently elicit humoral and cellular immunity.^35^ Moreover, mRNA vaccines have the potential to become personalized therapeutic drugs, in which they have application prospects for targeting specific tumors^37^ and treating rare diseases. Nevertheless, mRNA is susceptible to degradation by nucleases^33^ and suffers from thermal instability, necessitating a cold-chain infrastructure for storage and transportation, thereby increasing the overall cost.^38^ Furthermore, mRNA, being a large polyanionic structure, presents challenges in traversing cell and tissue barriers.^33^ Overcoming these limitations necessitates the development of appropriate delivery strategies.

Two innovative mRNA structures have been developed for specific purposes, namely self-amplifying mRNA (saRNA)^39^ and circular mRNA (circRNA).^40^ saRNA can confer equivalent vaccine protection effects to those of non-replicating mRNA at a reduced dosage.^41–43^ The primary distinction between saRNA and linear non-replicating mRNA is that saRNA contains additional self-amplifying replicon genes that originate from multiple positive-strand RNA viruses,^44–46^ resulting in a larger molecular size.^47^ Owing to the extensive anionic structure of saRNA, its delivery vehicle often comprises polycations. Nevertheless, the use of such delivery vectors may lead to high charge density that induces cytotoxicity. Hence, it is imperative to optimize a suitable delivery method for enhancing both the loading efficiency and the overall safety of saRNA drugs. Dastgerdi et al.^48^ demonstrated that optimizing the ratio of polyanions, such as γ-polyglutamic acid, to polycations in RNA formulations is an effective approach for improving the delivery efficiency and safety of saRNA. Furthermore, given the continuous generation of new mRNA within the body by saRNA vaccines, it is important to remain vigilant about safety concerns such as the potential for severe systemic or local inflammatory responses.^49^ CircRNA is a class of covalently closed-loop single-stranded RNA molecule that is not initiated by the classical translation pathway due to the absence of a 5’ cap and a 3’ tail.^50^ The internal loading of the ribosome may be the sole means for its translation to commence.^51^ The capability to encode proteins without the classical pathway is granted by circRNA’s ability to translate without a 5’ cap.^50^ Meanwhile, circRNA exhibits better structural and biochemical stability due to its inherent resistance against exonucleases-mediated degradation.^52^ Therefore, the average half-life of circRNA in cells significantly exceeds that of linear mRNA.^53^ Hence, cirRNA has the capacity to extend antigen production and sustain enduring immune responses.^54^ Nevertheless, despite the more persistent and robust expression of encoded proteins, circRNA seems to perform poorly in targeted delivery.^55^ At present, non-replicating IVT mRNA, which has been well-established in production and quality control, is primarily used in preclinical and clinical practice.

### Delivery system and preparation strategy of mRNA drugs

Due to the susceptibility of IVT mRNA to degradation by nucleases, it is crucial to select an appropriate delivery vehicle to safeguard the integrity of the mRNA prior to its intracellular delivery.^56^ An innovation initially not intended for mRNA but crucial for mRNA drug development is the advancement of delivery vectors, particularly the development of lipid nanoparticles (LNPs). At present, mRNA delivery systems can be classified into viral and nonviral systems.^57^

Virus-related delivery systems include both viruses and virus-like particles (VLPs).^58^ Viral vectors encompass adenoviruses, adeno-associated viruses (AAV), lentiviruses, herpes simplex virus (HSV), and Sendai virus.^59,60^ The formation of VLPs is facilitated by the structural proteins of a virus, which are viral components devoid of genetic material.^61^ In comparison to nonviral vectors, viral vectors possess the inherent advantage of efficiently entering cells and delivering nucleic acid drugs.^58^ This approach holds significant promise for vaccine development.^62^ Several clinical trials are currently utilizing viral vectors to deliver genetic material for the treatment of diseases. For instance, AAV9-mediated *CLN6* gene therapy (AT-GTX-501) has progressed to clinical stages I/II (NCT02725580), and interim findings indicated that AT-GTX-501 demonstrated favorable tolerability and could ameliorate the deterioration in motor and language function among pediatric patients with variant late infantile ceroid lipofuscinosis 6 (vLINCL6).^63^ Nevertheless, virus-mediated delivery systems pose potential biosecurity concerns (e.g., tumorgenicity and immunogenicity), exhibit low packaging efficiency, and entail high manufacturing costs.^59^ These limitations have driven researchers to investigate alternative delivery methods for mRNA vaccines, such as nonviral delivery systems.^62^

Various nonviral delivery vectors, such as LNPs,^64–68^ polymeric nanoparticles,^67,69,70^ lipid enveloped hybrid nanoparticles,^66^ protein/peptide-based nanoparticles,^67,68^ lyotropic liquid crystalline lipid nanoparticles (LCNPs),^71,72^ inorganic nanoparticles,^65^ nanoemulsions,^73,74^ exosomes,^64,75^ hydrogels,^76,77^ polymeric nanoparticle gel,^78^ and biological membrane-based vesicles,^79^ have been developed for efficient and versatile mRNA delivery. Optimizing the route of mRNA drug administration is essential for enhancing therapeutic efficacy, as it can significantly impact the biodistribution of the drug within tissues. Generally, intravenously injected mRNA drugs tend to accumulate in the liver, whereas locally administrated ones often prolong the duration of protein expression at the injection site and deliver sustained therapeutic effects. Subcutaneous and intramuscular injections represent the predominant routes for administering mRNA vaccines.^80^ Alternative mRNA drug delivery methods include aerosol inhalation to directly target the lungs,^81–83^ eye drops,^84^ intravitreal administration, subretinal administration,^80^ oral administration,^85,86^ transcutaneous route,^87^ and in utero delivery.^88^

Despite the development of various types of delivery vectors in preclinical studies, LNPs remain the established preferred delivery system for mRNA due to its clinical validation.^89^ Therefore, a comprehensive understanding of the design strategies employed in LNPs is essential for the effective implementation of mRNA therapies. The current formulation of LNPs for nucleic acid delivery typically comprises four key components: ionizable lipids, helper lipids, cholesterol, and polyethylene glycol (PEG)-lipids,^90,91^ with the ratio of ~50:10:38.5:1.5 mol%.^92^ Ionizable lipids play a crucial role in encapsulating mRNA and facilitating the successful transfection of mRNA into cells.^93^ The incorporation of PEG-lipids serves primarily to evade macrophage phagocytosis and ensure prolonged systemic circulation of LNPs. The inclusion of helper lipids and cholesterol allows for modulation of LNPs rigidity.^94^ Kulkarni et al.^95^ demonstrated that helper lipids and cholesterol play crucial roles in the formulation of LNPs, aiding in the encapsulation of genetic material.^96^ However, Su et al.^97^ broke away from the traditional LNPs model and discovered that cholesterol and phospholipids may not be essential for LNPs.

It is essential to elucidate the considerations for designing LNPs delivery systems, encompassing payload efficiency, stability, circulation time, facilitation of endosomal escape, biodegradability, immunogenicity, safety, and targeting capability.^98^ The precise structure of cationic lipids is pivotal, typically comprising a positively charged head and multiple hydrophobic tails. Even minor alterations, such as the addition or removal of an atom or a functional group, can significantly impact tissue targeting, cellular uptake, and endosomal escape.^99^ The design of ionizable lipids necessitates careful consideration of the pKa values of the headgroup in order to achieve an optimal pH-dependent electrostatic pattern.^98^ Ionizable lipids are typically positively charged in acidic pH environments and possess the capability to aggregate negatively charged mRNA into LNPs. At physiological pH, they become neutralized to minimize toxicity and enhance the biocompatibility of mRNA LNPs. Upon uptake by cells into acidic endosomes, they undergo protonation, thereby promoting the translocation of mRNA from endosomes into the cytosol.^93^ The pKa values of ionizable lipids can be modified by altering the chemical composition of the headgroup, such as imidazole, ester, and piperazine.^98^ The hydrophobic tails of the ionizable lipids play a crucial role in facilitating the assembly of LNPs and ensuring the stable encapsulation of mRNA. The design of the linker region between the headgroup and the lipid tails has a significant impact on the overall pH sensitivity of the ionizable lipids, consequently influencing both the release kinetics and magnitude of mRNA.^61^ The shape of ionizable lipids can also impact their functionality. More conical ionizable lipids, with a molecular structure that is not compatible with the lipid bilayer, can facilitate mRNA escape from lysosomes.^100^

The safety and stability concerns associated with mRNA therapy encompass various facets. With regard to safety, it is imperative to assess the biocompatibility of mRNA and its delivery system, minimize direct cellular damage, and mitigate the elicitation of unnecessary immune responses.^101^ However, the presence of pH-dependent or permanent cationic properties may result in safety concerns, such as disruption of cellular membranes and organelles, release of degradative enzymes from lysosomes, and damage to DNA. As a result, biocompatible molecular components capable of forming covalent ester or amide bonds have been developed for the production of biodegradable ionizable cationic lipids.^102^ In terms of stability, the carrier design should ensure protection of the mRNA from enzymatic degradation without impacting its release.^101^ In addition, enhancing the thermal stability of mRNA LNPs is crucial for reducing storage costs. For example, in order to enhance the thermostability of mRNA LNPs, researchers developed a novel ionizable lipid DOG-IM4 modified with imidazole.^103^

Modifying the physical and chemical characteristics of LNPs, such as their size, surface charge, and surface hydrophobicity, can impact their biological activity.^104^ Generally, hydrophilic LNPs with a neutral charge and a size smaller than 100 nm exhibit prolonged circulation in the bloodstream. LNPs with a positive charge are more conducive to cellular uptake.^105^ The morphology and nanostructure of LNPs are correlated with the transfection efficiency of mRNA. Nevertheless, the morphology of mRNA LNPs remains ambiguous, necessitating further research for elucidation.^106^ Furthermore, the precise localization of the four components within the LNPs remains unclear, posing certain limitations in molecular design.^107^ In order to tackle more demanding tasks for LNPs, such as targeted functionality, cellular penetration, and endosomal escape, the surface of LNPs can be subject to modification. Polymers like PEG-modified lipids are the predominant surface modifiers utilized for LNPs.^108^ Nevertheless, surface-modified LNPs may present potential adverse effects, such as allergic responses triggered by lipids modified with PEG.^61^ Furthermore, the challenges in scaling up the production of surface-modified LNPs could impede their commercialization.^108^

Presently, the commonly utilized methodologies for formulating LNPs encompass the thin-film hydration approach, solvent injection technique, reverse evaporation method, and microfluidic technology.^109^ Depending on the specific application, microfluidic chips can be fabricated from a variety of materials such as silicon, glass, polydimethylsiloxane (PDMS), cyclo-olefin polymers and copolymers (COPs/COCs). In addition, various fabrication methods including photolithography, electron beam lithography, wet and dry etching, and embossing can be employed. Microfluidic technology offers numerous advantages in the production of LNPs, facilitating continuous liquid flow within the microfluidic platform to ensure consistent nanoparticle quality over time and mitigate batch-to-batch variations. Furthermore, the design flexibility of microfluidic channels enables adaptation to different rapid mixing modes.^110^ Microfluidic technology has not only yielded remarkable results in laboratory research, but also been adopted and integrated into industrial production processes. The microfluidic device utilized in the preparation of BNT162b2 is an impingement jet mixer.^111^ Furthermore, ongoing advancements in automated high-throughput preparation techniques are continuously enhancing the production of LNPs^112^ and other nanodelivery vectors.^113^

## Preclinical and clinical applications of mRNA-based drugs

### Immunotherapy

mRNA vaccines outplayed their traditional counterparts in rapid development and cost-effectiveness trait,^114^ rendering them a promising tool for immunization against viruses, tumors, bacteria, and parasite. Despite the fact that mRNA was discovered in 1961, the revolutionary breakthrough of using it as a vaccine has only occurred in recent years, due to its susceptibility to enzymatic degradation, inefficient in vivo delivery, and intrinsic immunogenicity.^115^

In order to achieve antiviral, antitumor, antibacterial, and antiparasitic effects, mRNA-based immunotherapies must elicit a robust and specific host immune response to eliminate pathogens and confer durable protection.^28^ mRNA vaccines encoding disease-specific antigens function by transfecting antigen-presenting cells (APCs). The protein antigen is translated within the cell and released into the extracellular space or degraded by the proteasome to expose the antigenic sites. Intrinsic antigens can be presented by the cell surface major histocompatibility complex I (MHC I) to induce maturation of cluster of differentiation (CD) 8^+^ T cells, while extracellular antigen proteins can be recognized by B cells, internalized, degraded by APC cells, and presented through the MHC II class pathway to activate CD4^+^ T helper (Th) cells.^28,116^

In addition to disease-specific antigens, proteins related to immunotherapy encompass antibodies, cytokines, ligands, tumor suppressor proteins, and other functional proteins. It is worth noting that mRNA therapies encoding cytokines could be utilized to either enhance or suppress the immune response. Cytokines are a class of proteins that modulate immune cells by activating downstream cytokine receptors.^117^ Stimulatory cytokines are suitable for combating infectious diseases and cancer, while inhibitory cytokines are appropriate for treating autoimmune diseases. It is noteworthy that certain cytokines, such as Interleukin (IL)-2, do not have a fixed function in regulating the immune response; rather, their function changes with variations in concentration.^118,119^ Moreover, mRNA-based adoptive cell therapies may not be exclusively utilized for immune modulation or suppression, contingent upon the protein encoded by the mRNA. For instance, BNT211 is employed in the treatment of CLDN6-positive advanced solid tumors,^120^ while Descartes-08 is designated for autoimmune disorders.^121^

**Fig. 2:**
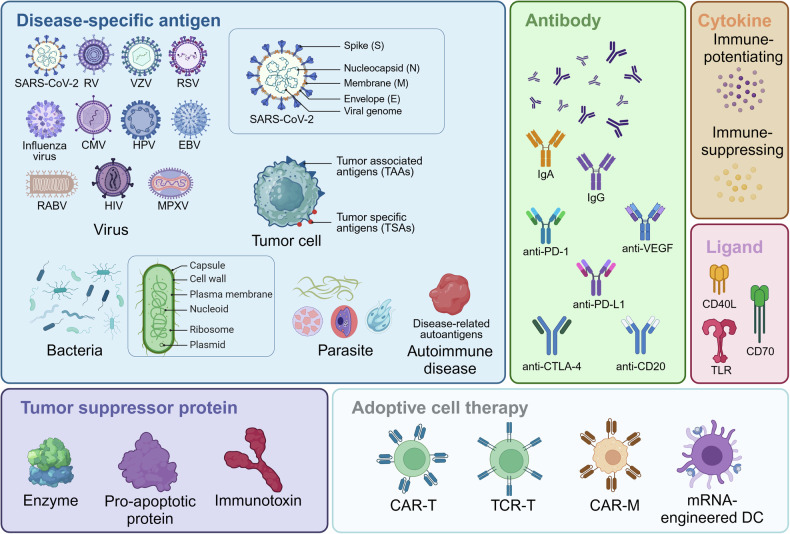
mRNA codes for immunotherapy-associated antigen, antibody, cytokine, ligand, tumor suppressor protein, and adoptive cell therapy. RV rotavirus, VZV varicella-zoster virus, RSV respiratory syncytial virus, CMV cytomegalovirus, HPV human papillomavirus, EBV Epstein–Barr virus, RABV rabies virus, HIV human immunodeficiency virus, MPXV monkeypox virus, anti-VEGF anti-vascular endothelial growth factor. The graphic is created with BioRender.com

In general, mRNA possesses the capacity to encode diverse proteins including antigens, antibodies, ligands, and tumor suppressor proteins. In addition, mRNA can also be harnessed in combination with adoptive cell therapy (Fig. 2), thus showcasing immense potential within the realm of immunotherapy (Table 1).

**Table 1:** Representative completed and ongoing clinical studies (immunotherapy)

Therapy	Subclass	mRNA drug	Application	mRNA-encoded protein	Delivery system	Administration route	Study started	Phase	Status	Result	NCT number	Sponsor	Ref.
Disease-specific antigen therapy	Virus antigen	mRNA-1273	COVID-19	Prefusion stabilized full-length spike protein	LNPs (SM-102, PEG2000-DMG, DSPC, cholesterol)	Intramuscular injection	2020-07-27	Phase III	Completed	Show 94.1% efficacy in preventing COVID-19 disease, with no safety concerns found except for transient local or systemic reactions	NCT04470427	ModernaTX, Inc.	^486^
		BNT162b2	COVID-19	SARS-CoV-2 full-length spike protein	LNPs (ALC-0315, ALC-0159, DSPC, cholesterol)	Intramuscular injection	2020-04-29	Phase II/III	Completed	Good immune efficacy against COVID-19 with a sustained safety profile and acceptable adverse events, but the immune efficacy declines after 6 months	NCT04368728	BioNTech SE	^487^
		mRNA-1345	RSV	RSV prefusion stabilized F (preF) glycoprotein	LNPs (ionizable lipid, phospholipid, PEG lipid, sterol)	Intramuscular injection	2023-10-06	Phase III	Recruiting	/	NCT06067230	ModernaTX, Inc.	^479^
		mRNA-1010	Seasonal influenza	Membrane-bound hemagglutinin (HA) surface glycoproteins of four influenza strains (A/H1N1, A/H3N2, B/Victoria, and B/Yamagata)	LNPs	Intramuscular injection	2022-09-14	Phase III	Completed	Acceptable safety and tolerability support continued study	NCT05566639	ModernaTX, Inc.	^134,488^
		mRNA-1647	CMV	Two CMV antigens (glycoprotein B and the pentameric glycoprotein complex)	LNPs	Intramuscular injection	2021-10-26	Phase III	Active, not recruiting	/	NCT05085366	ModernaTX, Inc.	^489^
		mRNA-1325	Zika virus	Premembrane and envelope E structural proteins (prME) from a Micronesia 2007 Zika virus isolate	LNPs	Intramuscular injection	2016-12-21	Phase I	Completed	Well tolerated, but poor Zika virus-specific nAb responses	NCT03014089	ModernaTX, Inc.	^490^
		mRNA-1893	Zika virus	prME from the RIO-U1 Zika virus isolate	LNPs	Intramuscular injection	2019-07-30	Phase I	Completed	Well tolerated, induce strong Zika virus-specific serum nAb responses after two doses that supported the continued study of mRNA-1893	NCT04064905	ModernaTX, Inc.	^490^
		mRNA-1388	CHIKV	Full CHIKV structural polyprotein (capsid and envelope proteins E3, E2, 6 k/TF, and E1) from CHIKV West African strain 37,997	LNPs (cholesterol, DPSC, ionizable lipid MC3, PEG2000-DMG)	Intramuscular injection	2017-08-15	Phase I	Completed	Good safety and immunogenicity	NCT03325075	ModernaTX, Inc.	^491^
		H10N8 mRNA vaccine (VAL-506440)	Influenza (H10N8)	Full-length, membrane-bound form of the HA glycoprotein from the H10N8 influenza strain (A/Jiangxi-Donghu/346/2013)	LNPs	Intramuscular injection	2015-12	Phase I	Completed	Well tolerated, trigger strong humoral immune responses	NCT03076385	ModernaTX, Inc.	^492^
		H7N9 mRNA vaccine (VAL-339851)	Influenza (H7N9)	Full-length, membrane-bound form of the HA glycoprotein from the H7N9 influenza strain (A/Anhui/1/2013)	LNPs	Intramuscular injection	2016-05-11	Phase I	Completed	Well tolerated, trigger strong humoral immune responses	NCT03345043	ModernaTX, Inc.	^492^
		CV7201	Rabies	Rabies virus glycoprotein	Cationic protein protamine as stabilizer and adjuvant	Intradermal, intramuscular injection	2013-10	Phase I	Completed	Generally safe and reasonably tolerated	NCT02241135	CureVac	^13^
		CV7202	Rabies	Rabies virus glycoprotein	LNPs (cholesterol, DSPC, PEGylated lipid, cationic lipid)	Intramuscular injection	2018-10-12	Phase I	Completed	Low doses (1 μg or 2 μg) were well-tolerated, whereas the 5 μg dose exhibited unacceptable reactogenicity	NCT03713086	CureVac	^493^
		mRNA-1644	HIV	eOD-GT8 60mer	Self-assembling nanoparticles	Intramuscular injection	2022-05-25	Phase I	Active, not recruiting	/	NCT05414786	International AIDS Vaccine Initiative	^156^
		mRNA-1644v2-Core	HIV	Core-g28v2 60mer	LNPs	Intramuscular injection	2021-11-12	Phase I	Active, not recruiting	/	NCT05001373	International AIDS Vaccine Initiative	^494,495^
		BNT166a	MPXV	MPXV antigens A35, B6, H3, and M1	LNPs	Intramuscular injection	2023-09-21	Phase I	Recruiting	/	NCT05988203	BioNTech SE	^167^
		EBV mRNA vaccine	EBV-positive advanced malignant tumors	Undisclosed	Undisclosed	Intramuscular injection	2022-11-18	Phase I	Recruiting	/	NCT05714748	West China Hospital	^496^
		BNT113	HPV16 positive head and neck squamous cell carcinoma (HNSCC) that expresses PD-L1	HPV16 oncoproteins E6 and E7	Liposomal	Intravenous injection	2021-01-07	Phase II	Recruiting	Acceptable safety profile	NCT04534205	BioNTech SE	^497^
		HBV mRNA vaccine	HBV-associated refractory hepatocellular carcinoma	HBsAg	LNPs	Intramuscular injection	2023-02-15 (estimated)	Phase I	Recruiting	/	NCT05738447	West China Hospital	^498^
	Tumor antigen	BNT111	Melanoma	Melanoma TAAs: New York esophageal squamous cell carcinoma 1 (NY-ESO-1), tyrosinase, melanoma-associated antigen 3 (MAGE-A3), and transmembrane phosphatase with tensin homology (TPTE)	Lipoplexes	Intravenous administration	2015-03	Phase I	Completed	Strong immunogenicity and promising clinical activity	NCT02410733	BioNTech SE	^174^
		BNT112	Prostate cancer	Prostate cancer TAAs: kallikrein-2, kallikrein-3, acid phosphatase prostate, homeobox B13 (HOXB13), and NK3 homeobox 1	Lipoplexes	Intravenous bolus injection	2019-12-19	Phase I/II	Terminated	Acceptable safety profile, induce robust prostate antigen-specific immune responses in patients with advanced prostate cancer	NCT04382898	BioNTech SE	^499^
		BNT116	Non-small cell lung cancer	Six shared antigens frequently expressed in non-small cell lung cancer	Undisclosed	Intravenous injection	2022-06-17	Phase I	Recruiting	/	NCT05142189	BioNTech SE	^500^
		mRNA-4157	Melanoma	Up to 34 patient-specific tumor neoantigens	LNPs	Intramuscular injection	2019-07-18	Phase IIb	Recruiting	Significantly extended distant metastasis-free survival in patients with resected high-risk melanoma as compared with Pembrolizumab monotherapy	NCT03897881	ModernaTX, Inc.	^481^
		mRNA-4157	Melanoma	Up to 34 patient-specific tumor neoantigens	LNPs	Intramuscular injection	2023-07-19	Phase III	Recruiting	/	NCT05933577	Merck Sharp & Dohme LLC	^501^
		Autogene cevumeran (RO7198457)	Pancreatic cancer	Neoantigen	Lipoplex nanoparticles	Intravenous delivery	2019-12-13	Phase I	Active, not recruiting	Preliminarily shown to be safe in combination with Atezolizumab and mFOLFIRINOX and to delay recurrence in patients with surgically removed pancreatic ductal adenocarcinoma (PDAC)	NCT04161755	Memorial Sloan Kettering Cancer Center	^502^
		Personalized tumor neoantigen mRNA vaccine	Liver cancer	Personalized tumor neoantigen	LNPs	Subcutaneous injection	2023-04-20	Not applicable	Not yet recruiting	/	NCT05761717	Shanghai Zhongshan Hospital	^503^
		Tumor neoantigen mRNA vaccine	Advanced esophageal cancer and non-small cell lung cancer	Tumor neoantigen	LNPs	Subcutaneous injection	2019-10-18	Not applicable	Recruiting	/	NCT03908671	Stemirna Therapeutics	^504^
		GRT-C901 and GRT-R902	Solid tumors	Neoantigen (#samRNA)	Chimpanzee adenovirus vector	Intramuscular injection	2019-02-13	Phase I/II	Completed	Well tolerated, induce neoantigen-specific CD8^+^ T-cell response in all patients, subsequent Phase II/III initiated (NCT05141721)	NCT03639714	Gritstone bio, Inc.	^505^
	Bacterial antigen	mRNA-1975 and mRNA-1982	Lyme disease	Undisclosed	Undisclosed	Intramuscular injection	2023-07-26	Phase I/II	Active, not recruiting	/	NCT05975099	ModernaTX, Inc	^188^
		BNT164a1 and BNT164b1	Tuberculosis	Undisclosed	Undisclosed	Intramuscular injection	2023-07-31	Phase I/II	Recruiting	/	NCT05547464	BioNTech SE	^453^
	Parasite antigen	BNT165b1	Malaria	Part of the Plasmodium falciparum circumsporozoite protein (PfCSP)	LNPs	Intramuscular injection	2022-12-15	Phase I	Active, not recruiting	/	NCT05581641	BioNTech SE	^201^
Therapeutic antibody therapy	Antibody	mRNA-1944	CHIKV	Light and heavy chains of a human monoclonal antibody (CHKV-24 IgG) targeting the CHIKV E2 glycoprotein	LNPs (a proprietary IAL; a proprietary high-purity PEG-2k-stearate monoester; cholesterol, DOPE)	Intravenous infusion	2019-01-22	Phase I	Completed	Acceptable safety profile, produce high levels of functionally neutralizing antibodies	NCT03829384	ModernaTX, Inc.	^506^
		BNT141	Unresectable or metastatic CLDN18.2-positive gastric, pancreatic, ovarian and biliary tract tumors	Anti-CLDN18.2 antibodies	LNPs	Intravenous injection	2022-01-18	Phase I/II	Terminated	/	NCT04683939	BioNTech SE	^507^
		BNT142	CLDN6-positive solid tumors	T-cell-engaging bispecific antibody against CLDN6 and the T-cell receptor–associated molecule CD3	LNPs	Intravenous bolus/infusion	2022-03-28	Phase I/II	Recruiting	/	NCT05262530	BioNTech SE	^508^
		RNA-transfected mature autologous DC	Melanoma	Anti-CTLA-4 and anti-GITR mAb and melanoma TAAs MART, tyrosinase, and gp100, and MAGE-3	DCs	Intranodal injection	2010-01	Phase I	Terminated	/	NCT01216436	Duke University	^236^
Cytokine	Immune-potentiating	MEDI1191	Solid tumors	IL-12	LNPs	Intratumoral injection	2019-05-08	Phase I	Completed	/	NCT03946800	MedImmune LLC	^259^
		mRNA-2752	Advanced solid tumors and lymphoma	OX40L, IL-23, and IL-36γ	LNPs	Intratumoral injection	2018-11-27	Phase I	Active, not recruiting	Tolerated, may be associated with tumor shrinkage	NCT03739931	ModernaTX, Inc.	^509^
		SAR441000	Solid tumors	IL-12 single chain, IFN ɑ-2b, GM-CSF, and IL-15 sushi	Saline-formulated mixture	Intratumoral injection	2019-01-03	Phase I	Terminated	Well tolerated (clinical trial in 17 patients; July 2020), supporting further clinical studies	NCT03871348	Sanofi	^263^
		BNT153 and BNT152	Solid tumors	IL-2, IL-7	LNPs	Intravenous	2021-06-08	Phase I	Recruiting	/	NCT04710043	BioNTech SE	^510^
	Immune-suppressing	mRNA-6231	Healthy volunteers	Human serum albumin IL-2 mutein fusion protein (HSA-IL2m)	LNPs	Subcutaneous injection	2021-07-28	Phase I	Completed	/	NCT04916431	ModernaTX, Inc.	^511^
Ligand	Ligand	TriMix	Breast cancer	DC-activating [CD40 ligand (CD40L), CD70, and constitutively active Toll-like receptor 4 (TLR4)] proteins	Undisclosed	Intratumoral administration	2018-11-12	Phase I	Recruiting	/	NCT03788083	Universitair Ziekenhuis Brussel	^512^
Adoptive cell therapy	CAR-T	CLDN6 CAR-T cells +/− CARVac	CLDN6-positive advanced solid tumors	CLDN6	Lipoplexes	Intravenous injection	2020-09-16	Phase I/II	Recruiting	Good safety profile and encouraging efficacy	NCT04503278	BioNTech Cell & Gene Therapies GmbH	^120^
	CAR-T	Descartes-08	Myasthenia gravis	Anti-BCMA targeting CAR protein	Autogolous T cells	Infusion	2019-12-04	Phase Ib/IIa	Recruiting	Safe and well-tolerated	NCT04146051	Cartesian Therapeutics	^121^
	TCR-T	LioCyx-M	Recurrent hepatocellular carcinoma	HBV-specific TCR	Autologous T cells	Infusion	2015-07-02	Phase I	Unknown status	Well tolerated, have no adverse effects on the transplanted liver	NCT02719782	Lion TCR Pte. Ltd.	^513^
	CAR-PBMC	MCY-M11	Advanced ovarian cancer and peritoneal mesothelioma	Anti-mesothelin CAR (Meso-CAR)	PBMCs	Intraperitoneal infusion	2018-08-27	Phase I	Terminated	Mediate effective and long-term antitumor response	NCT03608618	MaxCyte, Inc.	^329^
	DCs	AGS-004	HIV	CD40L and three or four autologous HIV-1 antigens	DCs	Undisclosed	2008-02	Phase II	Completed	/	NCT00672191	Argos Therapeutics	^514^
		WT1 antigen-targeted DC vaccine	Acute myeloid leukemia (AML)	WT1 antigen mRNA loaded autologous DCs (by electroporation)	DCs	Undisclosed	2012-10	Phase II	Active, not recruiting	/	NCT01686334	Zwi Berneman	^515^
		DC vaccine (DCs transfected with Survivin, hTERT and p53 mRNA)	Metastatic breast cancer or malignant melanoma	Survivin, hTERT and p53	DCs	Intradermal injection	2009-09	Phase I	Completed	/	NCT00978913	Inge Marie Svane	^516^
		Human CMV pp65-LAMP mRNA-pulsed autologous DCs	GBM	CMV protein pp65	DCs	Intradermal and bilateral administration at the groin site (divided equally to both inguinal regions)	2015-10-12	Phase II	Completed	Effective GBM inhibition	NCT02366728	Mustafa Khasraw, MBChB, MD, FRCP, FRACP	^517^

*LNPs* lipid nanoparticles, *PEG* polyethylene glycol, *DSPC* distearoylphosphatidylcholine, *RSV* respiratory syncytial virus, *CMV* cytomegalovirus, *CHIKV* chikungunya virus, *HIV* human immunodeficiency virus, *MPXV* monkeypox virus, *EBV* Epstein–Barr virus, *HPV* human papillomavirus, *HBV* hepatitis B virus, *TAAs* tumor-associated antigens, *DOPE* 1,2-dioleyl-sn-glycero-3-phosphoethanolamine, *CLDN6* claudin 6, *DCs* dendritic cells, *IL* interleukin, *IFN* interferon, *GM-CSF* granulocyte-macrophage colony-stimulating factor, *TriMix* mRNA-encoding CD40L, CD70, and TLR4, *CAR* chimeric antigen receptor, *BCMA* B-cell maturation antigen, *TCR* T-cell receptor, *PBMC* peripheral blood mononuclear cells, *WTI* Wilms’ tumor, *GBM* glioblastoma

#### Disease-specific antigen therapy

##### Virus antigen mRNA vaccine

**Fig. 3:**
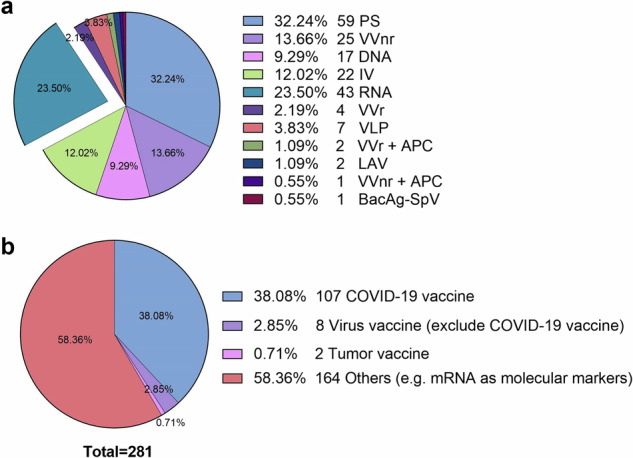
Application of mRNA vaccine in immunotherapy. **a** Types of COVID-19 vaccines in clinical trials (from WHO; March 30, 2023). **b** mRNA-based drugs in Phase III/IV trials. On clinicaltrial.gov, search for “mRNA” as the only keyword and the search criteria are limited to “Phase 3” and “Phase 4”. Note that this search method cannot find all mRNA vaccines, and some mRNA vaccines do not contain the word “mRNA”, so the figure should be viewed dialectically. PS protein subunit, VVnr viral vector (non-replicating), IV inactivated virus, VVr viral vector (replicating), VLP virus-like particle, VVr + APC VVr + antigen-presenting cell, LAV live attenuated virus, VVnr + APC VVnr + antigen-presenting cell, BacAg-SpV bacterial antigen-spore expression vector

**SARS-CoV-2:** In 2019, the novel coronavirus emerged globally, leading to widespread transmission and the tragic loss of numerous lives, thereby posing a huge threat to global health and sanitation. The scarcity of a specific therapeutic agent for post-infection treatment with SARS-CoV-2 caused global panic. In response to this challenge, a variety of SARS-CoV-2 vaccines have been propelled into an intense and accelerated research phase. mRNA vaccines, representing an innovative technology for the prevention of infectious diseases, were initially anticipated to require 5–6 years to reach the market.^122^ Encouragingly, following the public disclosure of the coronavirus RNA sequence in January 2020,^122^ it took less than 3 months for the mRNA vaccines, mRNA-1273 (NCT04283461, Phase I initiated in March 2020) and BNT162b2 (NCT04380701, Phase I and Phase II initiated in April 2020), to transition from development to clinical trials.^123^ Remarkably, within a year, both mRNA vaccines (BNT162b2 and mRNA-1273) rose to prominence, securing emergency approval from the U.S. FDA and were authorized for global mass vaccination against SARS-CoV-2.^122,124^ According to the consolidated information provided by the World Health Organization (WHO), as of March 30, 2023 (Fig. 3a), a total of 183 vaccines had entered the clinical stage, with RNA-based vaccines accounting for about 24% (43 in total), ranking second only to protein subunit vaccines which account for around 32% (59 in total).^125^

**Rotavirus:** The successful application of mRNA vaccines against COVID-19 disease has prompted researchers to swiftly expand them to other antiviral fields. Rotaviruses (RVs) are responsible for causing diarrhea in children worldwide and can result in subsequent gastroenteritis, yet there is no specific treatment for this virus. Lu et al.^14^ designed an mRNA vaccine encoding the VP7 protein, which is one of the capsid proteins of RVs and plays an important role in the infection of target cells, for preventing RVs infection. The VP7 mRNA vaccine was inoculated to mice via intramuscular or subcutaneous injection (three doses). The findings demonstrated that the VP7 mRNA vaccine effectively stimulated T-cell immune responses and elicited RV-specific antibodies.

**Varicella-zoster virus:** Varicella-zoster virus (VZV), a member of the alphaherpesvirus subfamily, is a neurotropic human herpesvirus.^126^ The initial infection manifests as chickenpox symptoms, after which the virus remains latent in the body. Reactivation of the virus occurs when the immune system weakens due to aging or compromised immunity.^127^ In 2024, Huang et al.^128^ developed an mRNA-based VZV vaccine called ZOSAL, which employed ionizable LNPs for encapsulating sequence-optimized mRNA encoding the full-length glycoprotein E. In this study, the immunogenicity, safety, and immune mechanisms of ZOSAL were compared with those of the licensed protein-based vaccine Shingrix in mice and rhesus macaques. The findings demonstrated that ZOSAL exhibited superior immunogenicity, safety profile, and capacity to induce virus-specific T-cell immunity when compared to Shingrix.

**Respiratory syncytial virus:** Respiratory syncytial virus (RSV) is an enveloped, single-stranded RNA virus, which can cause respiratory illnesses.^129^ RSV can result in fatal outcomes for pediatric, geriatric, and immunocompromised individuals.^130^ One of the highly progressive vaccines aimed at combating RSV is mRNA-1345, which is presently being developed by ModernaTX, Inc. This vaccine encodes the membrane-anchored RSV prefusion stabilized F (preF) glycoprotein.^131^ Currently, the mRNA-1345 vaccine for RSV is undergoing Phase III trial (NCT05330975, NCT06067230), and it has already received FDA approval via fast-track designation for administration in individuals aged 60 years and older.^132^

**Influenza virus:** Influenza viruses have the ability to infect a wide range of vertebrates and are responsible for causing seasonal influenza as well as influenza pandemics in humans.^133^ ModernaTX, Inc. developed a quadrivalent mRNA vaccine (mRNA-1010) targeting seasonal influenza, encoding the four hemagglutinin (HA) surface glycoproteins of influenza strains (A/H1N1, A/H3N2, B/Victoria, and B/Yamagata). The vaccine has undergone testing in healthy adults during Phase I/II trial (NCT04956575), with interim results demonstrating favorable safety.^134^ Further, it has currently enrolled 22,510 adults aged 50 years and older (NCT05566639)^135^ and 8400 adults aged 18 years and older (NCT05827978)^136^ to initiate Phase III safety and efficacy studies.

**Cytomegalovirus:** Cytomegalovirus (CMV) is an incredibly prevalent virus, with estimated incidence rates of 60% in developed countries and 90% in developing countries. Although asymptomatic in healthy individuals, infection with CMV can pose a significant risk to immunocompromised patients (such as those undergoing organ transplantation), potentially leading to graft rejection and even life-threatening complications. In addition, it is imperative to acknowledge the issue of vertical transmission of CMV from pregnant women to fetuses, which can result in premature birth and profound permanent disabilities in neonates, encompassing cognitive impairment and visual deficits. In order to tackle this concern, ModernaTX, Inc. developed an mRNA vaccine (mRNA-1647) that encodes two CMV proteins, namely glycoprotein B (gB) and pentameric gH/gL/UL128/UL130/UL131A glycoprotein complex (pentamer). The vaccine has successfully completed Phase I clinical trial (NCT03382405), demonstrating that administration of three doses of mRNA-1647 (180 µg) could effectively elicit high titers of neutralizing antibodies (nAbs), broad neutralization activity, robust T-cell response, and long-lasting memory B cells in healthy adults,^137^ while maintaining an acceptable safety profile.^138^ Hu et al.^139^ compared mRNA-1647 (NCT03382405) with MF59-adjuvanted gB subunit (gB/MF59) vaccine (NCT00133497), and observed that although the gB-specific IgG responses were lower after inoculation with mRNA-1647 vaccine, it elicited persistent HCMV-specific antibody responses and higher antibody-dependent cellular cytotoxicity responses. In addition, a Phase III trial was conducted in healthy female participants aged 16–40 years with mRNA-1647 (NCT05085366).^140^

**Human papillomavirus:** The distinctive properties of certain viruses, such as carcinogenicity, render mRNA vaccines encoding viral proteins a promising strategy for combating tumors. HPV infection is widely recognized as the primary factor contributing to cervical cancer.^141,142^ The E6 and E7 proteins of human papillomavirus (HPV) have been established to modulate cell cycle and exhibit a high association with cervical cancer. In light of this, Lee et al.^143^ designed an mRNA vaccine encoding the E6 and E7 proteins, which was subsequently administered to C57BL/6J mice via intramuscular or subcutaneous injection. Their findings revealed that the mRNA vaccine markedly elicited robust T-cell-mediated immune responses in tumor-bearing mice, leading to a significant inhibition of tumor growth. Importantly, the experimental results demonstrated that the vaccine not only exhibited therapeutic potential against existing tumors but also displayed preventive efficacy.

**Epstein–Barr virus:** Epstein–Barr virus (EBV), an oncogenic virus in humans, is frequently associated with nasopharyngeal carcinoma (NPC). LMP2 is one of the main viral proteins. Xiang et al.^144^ delivered LMP2-mRNA LNPs to tumor-draining lymph nodes (TDLN) for the purpose of inducing activation and cytotoxicity of CD8^+^ T cells against tumor cells expressing LMP2. Zhao et al.^145^ developed three therapeutic EBV mRNA vaccines that encoded truncated latent EBV protein regions containing abundant T-cell epitopes, including truncated forms of latent membrane protein 2A (LMP2A), EBV nuclear antigen 1 (EBNA1), and EBV nuclear antigen 3A (EBNA3A). Their findings demonstrated that these EBV mRNA vaccines could effectively elicit antigen-specific immune responses, thereby suppressing tumor progression and prolonging the survival time of tumor-bearing mice.

**Rabies virus:** The neurotropic rabies virus (RABV), a member of the Lyssavirus family and a single-stranded RNA virus,^146^ has the ability to infect all warm-blooded animals,^147^ including humans, leading to the development of rabies. Rabies is a fatal neurological disease, and once the initial symptoms manifest, there is minimal time to pursue treatment options, as the mortality rate approaches 100%.^146^ Rabies causes an estimated 59,000 fatalities annually on a global scale.^148^ The currently most widely used rabies vaccines are all inactivated, requiring individuals to receive three to five doses for optimal immune protection.^149,150^ In summary, despite advancements in rabies vaccine development, the current availability of vaccines is constrained by factors such as high costs, limited production capacity and storage requirements, as well as the necessity for multiple doses.^147^ The mRNA vaccines represent a promising approach that has the potential to reduce costs and lower the required dosage for achieving effective immune protection.^150^ RABV glycoprotein (RABV-G), being the only virion-surface protein of RABV, plays a crucial role as an antigen in vaccine development.^151^ In 2013, CureVac conducted the initial Phase I clinical trial for an mRNA-based rabies vaccine in healthy adults (NCT02241135). The trial findings indicated that the mRNA vaccine (CV7201) encoding the RABV-G demonstrated a favorable safety profile.^13^ Subsequently, CureVac also progressed a mRNA vaccine named CV7202, encoding the RABV-G protein, into Phase I clinical trial (NCT03713086). Currently, research on the mRNA vaccine for RABV-G is ongoing. Cao et al.^152^ developed a novel mRNA LNPs that encodes RABV-G with the H270P mutation, which can stabilize the prefusion conformation of RABV-G. Their findings demonstrated that this vaccine elicited superior humoral and cellular immune responses compared to mRNA LNPs encoding RABV-G, indicating that structured-guided vaccine design may be the future direction for vaccine development.

**Human immunodeficiency virus:** Acquired immunodeficiency syndrome (AIDS), resulting from infection with the human immunodeficiency virus (HIV), remains a fatal disease for humans.^153^ Although current antiretroviral therapies^154^ and therapeutic vaccines can improve HIV-related morbidity and mortality, they are still unable to completely eradicate HIV.^155^ At present, the HIV mRNA vaccines that have been developed and entered clinical trials include the eOD-GT8 60mer mRNA vaccine (mRNA-1644, NCT05414786)^156^ and the Core-g28v2 60mer mRNA vaccine (mRNA-1644v2-Core, NCT05001373).^157^ As of June 2024, these vaccines are still undergoing Phase I clinical trials. Despite the evaluation of various HIV vaccines in preclinical and clinical studies, the overall outcomes have been unsatisfactory.^158^ Further endeavors are imperative for the development of an effective HIV vaccine. Mandal et al.^159^ designed LNPs containing mRNA that encoded multiple epitopes of HIV viral protease cleavage sites. The mRNA LNPs exhibited long-term stability at cold-chain temperatures and could elicit potent cellular immunity, making it as a promising candidate for a prophylactic HIV mRNA vaccine. A major obstacle in the eradication of HIV lies in the virus’s ability to conceal itself within host cells, including myeloid cells and CD4^+^ T cells.^160^ It appears that vaccines alone may not be sufficient for curing HIV, prompting consideration of combining vaccines with other treatment modalities.^155,160^ The extensive genetic diversity of HIV and its capacity to integrate into the host cell genome undoubtedly pose significant challenges for the development of HIV vaccines.^161,162^

**Monkeypox virus:** Monkeypox is a communicable disease caused by the monkeypox virus (MPXV), which can be transmitted between humans and animals.^163,164^ MPXV is a type of double-stranded DNA virus^163,165^ that belongs to the *Orthopoxvirus* genus.^165,166^ Currently, a multivalent mRNA vaccine (BNT166) encoding MPXV antigens A35, B6, H3, and M1 has entered Phase I clinical trial (NCT05988203)^167^. Several preclinical studies are underway to develop novel mRNA vaccines targeting various antigens of the virus. Tian et al.^168^ designed an mRNA-based vaccine encoding the MPXV A29L antigen. Su et al.^169^ developed a quadrivalent mRNA vaccine that encoded the antigens A27, L1, A33, and B5 of vaccinia virus. It is noteworthy that the sera from mice immunized with this vaccine demonstrated reactivity with the antigens of various orthopoxviruses in vitro. This suggests that the mRNA vaccine holds promise for safeguarding humans against MPXV and other orthopoxvirus infections.

##### Tumor antigen mRNA vaccine

Cancer is a major cause of global mortality.^170^ The inception of antitumor mRNA vaccines can be traced back to 1995, when Conry et al.^171^ constructed an mRNA encoding the tumor antigen–human carcinogenic antigen (CEA) that could be directly injected into the skin to combat tumors in mouse models. In 2004, a clinical trial was initiated to intradermally administer mRNA-encoding melanoma-associated antigens to stage III/IV melanoma patients (NCT00204607).^9^ In 2009, Weide et al.^10^ reported the results of the Phase I/II trial, demonstrating that the feasibility and safety of direct injection of protamine-protected mRNA, thereby encouraging further clinical studies on mRNA vaccines.

A critical step in the development of cancer vaccines involves the identification and selection of appropriate tumor antigens, encompassing both TAAs and tumor-specific antigens (TSAs).^172^

TAAs are non-mutated proteins that exhibit high expression in tumors while showing no-to-low expression in normal tissues.^173^ The RNA-LPX vaccine (BNT111), which has successfully completed Phase I clinical trial (NCT02410733) and is now progressing to Phase II (NCT04526899), is a tumor vaccine designed to encode TAAs (New York esophageal squamous cell carcinoma 1, melanoma-associated antigen 3, tyrosinase, and transmembrane phosphatase with tensin homology) for melanoma.^174,175^ The results of the clinical trial demonstrated that the intravenous administration of BNT111 vaccine in patients can elicit a robust and broad T-cell response against TAAs.^117^ However, the use of TAAs as tumor antigens is limited by their expression in normal tissues to some extent, which may hinder the development of effective antitumor immune responses owing to the self-tolerance mechanisms and the potential for off-target effects that may lead to autoimmune toxicity.^173,176^

TSAs, which result from mutations in somatic cells, hold significant potential for inducing specific T-cell responses against tumors.^173^ For instance, Moderna’s mRNA-4157 is an mRNA vaccine that encodes up to 34 patient-specific tumor neoantigens.^177^ It became the first mRNA cancer vaccine to enter Phase III clinical trial (NCT05933577). The clinical IIb trial of mRNA-4157 in combination with Pembrolizumab treatment demonstrated a 49% reduction in the risk of mortality among high-risk melanoma patients.^176^ TSAs are promising targets for cancer vaccines.^178^ However, only a small fraction of somatic mutations in cancer cells could be recognized by spontaneously occurring T cells, and the efficacy of these neoepitopes in mediating antitumor effects varies, posing challenges for their precise utilization.^173^

To address the challenge of limited availability of targetable neoantigens and antigen target loss in tumors, it is imperative to investigate and utilize the full spectrum of tumor antigens, to develop personalized cancer vaccines. Trivedi et al.^179^ established an immunogenomics pipeline called “Open Reading Framework Antigen Network (O.R.A.N.)” to effectively identify immunogenic antigens with high likelihood of becoming therapeutic targets. In addition, they developed a platform called “Tumor Open reading Frames that are Unique (TOFU)” which utilizes IVT mRNA technology for encoding multiple tumor antigens into a single mRNA vaccine, thereby enabling the customization of virtually limitless quantities of antigens specific to each tumor type. Ben-Akiva et al.^180^ developed a class of bioreducible nanocarriers utilizing lipophilic poly(beta-amino ester) to encapsulate antigen-encoding mRNA and Toll-like receptor (TLR) agonist adjuvants for the treatment of murine melanoma and colon adenocarcinoma. Due to its mRNA sequence-independent encapsulation capacity, this platform can also be extended to address other cancer diseases.

The standalone mRNA cancer vaccine may serve as an effective treatment option for early-stage cancer. However, in the case of advanced cancer patients, the tumor microenvironment often exhibits a high level of immune suppression, thereby diminishing the efficacy of mRNA monotherapy.^181^ Instead, combining therapeutic cancer mRNA vaccines with other immunotherapies,^182^ such as the combination of mRNA-4157 and Pembrolizumab to treat high-risk melanoma, holds promise for success.^183^

Despite the fact that mRNA vaccines encoding tumor antigens entered Phase I trials earlier than viral mRNA vaccines, it is disappointing that few mRNA vaccines against cancer seem to have advanced to Phase III or IV trials (Fig. 3b).^184^ The reasons are summarized in “Conclusions and prospects” section. We anticipate an influx of approved drugs and methodologies for cancer treatment, instilling renewed optimism in patients.

##### Bacterial antigen mRNA vaccine

The conventional approach to eradicating bacteria involves the administration of antibiotics. However, due to the overuse of these drugs, we have been alarmed by the emergence of antibiotic-resistant bacteria and even the development of highly resistant strains. Nevertheless, it is important to note that the research and approval process for creating a new antibiotic is exceedingly time-consuming and necessitates substantial financial resources. Regrettably, bacteria can develop resistance at a much faster rate than new drugs can be developed. Consequently, despite our awareness regarding the imperative need for antibiotics control measures, we may find ourselves in a situation where no effective drug exists to combat bacterial infections. Therefore, it is essential to propose an innovative antimicrobial therapy. Given that mammalian organisms are capable of translating mRNA-encoding bacterial proteins, mRNA vaccines offer a promising and efficacious avenue for preventing bacterial diseases.^185,186^

Lyme disease is a prevalent infectious ailment in the U.S. that currently lacks a specific vaccine. Among various candidates, outer surface protein A (OspA) that expressed on the pathogen Borrelia burgdorferi has emerged as most promising antigen for developing a platform to combat Lyme disease. Therefore, Pine et al.^187^ developed a mRNA LNPs encoding OspA and observed that its protective efficacy surpassed that of the OspA protein subunit vaccine. The treatment regimen of mRNA-1975 and mRNA-1982 developed by ModernaTX, Inc. for Lyme disease is currently in Phase I/II clinical trial (NCT05975099).^188^

As a significant infectious disease, Tuberculosis (TB), caused by *Mycobacterium Tuberculosis* (M. TB),^189^ continues to present a grave global public health menace. Currently, the only licensed vaccine for TB is Bacillus Calmette–Guerin (BCG), which exhibits limited efficacy in preventing the disease.^190^ The absence of effective vaccine strategies and the emergence and dissemination of multidrug-resistant TB (MDR-TB) bacteria have further complicated endeavors to prevent and treat TB. Similar to COVID-19, TB also represents a respiratory infection. Can the mRNA vaccine strategy successfully employed for COVID-19 be replicated for TB?^191^ Fortunately, in 2023, BioNTech SE commenced Phase I/II clinical trial (NCT05547464) for an mRNA approach targeting TB (BNT164a1 and BNT164b1),^185,192^ and we expect a satisfactory therapeutic efficacy from this strategy.

Currently, the application of antibacterial mRNA vaccines in preclinical and clinical attempts is limited, possibly due to the intricate nature of bacterial infections compared to viral infections. Bacterial infections exhibit distinct molecular expression characteristics at different stages of infection, making it virtually impossible for a single antigen to effectively cover all these stages.^193^ Moreover, bacterial pathogens display diversity and the variability in antigen expression while also evolving multiple mechanisms to evade or suppress immune responses.^185^ Therefore, if mRNA vaccines are intended for further application in preventing and treating bacterial infections, careful consideration must be given to rational antigen selection along with the incorporation of adjuvants and other optimization strategies.^193^ The research conducted by Meulewaeter et al.^194^ demonstrated that the incorporation of adjuvant αGC into the *Listeria monocytogenes* mRNA LNPs resulted in a synergistic protective effect against listeriosis, underscoring the therapeutic advantages of co-activating invariant natural killer T (iNKT) cells in antibacterial mRNA vaccines. We are looking forward to the successful development of the first antibacterial mRNA vaccine product.

##### Parasite antigen mRNA vaccine

In addition to viral infections, parasitic diseases are also infectious illnesses that debilitate individuals. Parasitic diseases can be generally categorized into three groups: ectoparasitic, protozoal, and helminthic diseases. These conditions can be managed through proactive vector control, disease monitoring, the use of effective vaccines/medications, and the implementation of appropriate sanitation infrastructure.^195^ However, the lack of public awareness and the absence of appropriate treatment options have resulted in widespread parasitic infections.^196^

Malaria is the most deadly parasitic vector-borne disease^197^ caused by at least five species of *Plasmodium* parasites.^198–200^ Over the years, researchers have endeavored to develop vaccines targeting different stages of the malaria parasite’s life cycle. However, the only approved vaccine to date is Mosquirix, which is based on recombinant proteins. Nevertheless, Mosquirix has encountered several limitations, including suboptimal efficacy in specific age groups and the necessity for multiple booster shots to achieve a satisfactory level of protection. Fortunately, the mRNA vaccine (BNT165b1) developed by BioNTech, which encodes a segment of the *Plasmodium falciparum* circumsporozoite protein (PfCSP), has progressed to Phase I clinical trial (NCT05581641).^201^ Kunkeaw et al.^202^ developed the LNPs platform to deliver nucleotide-modified mRNA encoding the Pvs25 antigen of Plasmodium vivax, a potential vaccine against malaria transmission. Pvs25 is an antigen candidate for blocking malaria transmission. They discovered that the mRNA LNPs platform could elicit stronger and more enduring functional immunity compared to the Pvs25 recombinant protein vaccine. Additional mRNA vaccines against malaria are currently undergoing preclinical research.^203–205^

The lack of interest in the commercial development of parasite vaccines may be attributed to the fact that parasitic diseases primarily affect economically disadvantaged populations, and most parasites cause chronic illnesses that are non-lethal to the host, with the exception of the deadly malaria. Parasite infections are intricate, and the development of conventional vaccines entails high costs.^206^ In these regards, mRNA-based vaccines present a promising approach to address these challenges.

##### Autoimmune disease antigen therapy

The immune system plays a critical role in defending the body against infections caused by viruses, bacteria, fungi, and parasites. Additionally, it surveils and eliminates cancerous cells to maintain the body’s health. Autoimmune disorders arise when the immune system erroneously targets healthy tissues and cells in the body.^207,208^ An essential characteristic of autoimmune diseases is the presence of autoantibodies that have the ability to specifically bind to the antigens present in the patient’s healthy cells or tissues.^207,209^ There are more than 80 autoimmune disorders, including multiple sclerosis (MS),^210^ type 1 diabetes (T1D),^211^ systemic lupus erythematosus (SLE),^212^ inflammatory bowel disease (IBD),^213^ and rheumatoid arthritis (RA).^214^ The objective of treating autoimmune diseases is to regulate the activity of autoreactive cells in the body without inducing systemic immune suppression.^215^

Krienke et al.^210^ utilized a non-inflammatory lipid complex carrier to encapsulate *N*^1^-methyl-pseudouridine-modified mRNA (m^1^Ψ mRNA) encoding disease-related autoantigens. Their findings indicated that the use of this m^1^Ψ mRNA vaccine could stimulate antigen-specific regulatory T cells (Tregs) and mitigate the severity of multiple sclerosis in a mouse model.^216^ This strategy seems capable of inducing cross-tolerance in autoimmune T cells while preserving the normal immune response of the immune system.^217^ This also implied that by reducing unnecessary immunogenicity, mRNA vaccines encoding autoantigens can be a means to induce and sustain natural peripheral immune tolerance.^218^

Type 1 diabetes is an autoimmune condition in which the immune system mistakenly attacks and destroys the pancreatic islet β-cells, resulting in insufficient insulin production.^211,219^ Foster et al.^220^ discovered that the utilization of a novel mRNA vaccine encoding proinsulin II could transiently delay diabetes symptoms in nonobese diabetic mice. This effect may be attributed to the ability of the mRNA vaccine to restore immune tolerance in nonobese diabetic mice.

The mRNA therapies hold significant promise in the realm of autoimmune diseases.^221,222^ For instance, the mRNA drugs can be tailored to encode proteins or peptides capable of binding and neutralizing autoantibodies. In addition, mRNA can be engineered to specifically target plasma cells and express pro-apoptotic molecules within them, thereby selectively disrupting or eliminating their function and survival. Furthermore, given the pivotal role of Tregs in suppressing immune responses, mRNA can be designed to bolster the functionality of Treg cells.^207^

#### Therapeutic antibody therapy

Antibodies, also referred to as immunoglobulins, are effector molecules generated by the humoral immune responses that can neutralize antigens^223^ and play a crucial role in the prevention, control, and elimination of infections.^224^ The emergence of the therapeutic antibody can be traced back to 1975, when Köhler and Milstein pioneered the hybridoma technology and laid the foundation for the progress of monoclonal antibodies (mAbs).^225^ Currently, therapeutic antibodies have emerged as a potent armamentarium against a variety of diseases,^223^ demonstrating remarkable efficacy in treating viral illnesses, solid tumors, hematological malignancies, autoimmune disorders, and other indications. In the last few decades, antibodies have experienced unprecedented growth as new drugs for these indications.^226^

However, protein-based drugs such as mAbs encounter challenges in terms of production and cost, including intricate manufacturing process, high purification expenses, and difficulties in post-translational modification of proteins. In addition, the human body harbors a total of 22 distinct amino acids, and due to variations in their types, quantities, and arrangements, proteins can exhibit diverse physicochemical properties. To ensure protein storage stability, specialized buffer solutions optimized for this purpose are indispensable.^227^ The nucleic acids, in contrast, carry a negative charge and exhibit similar physicochemical characteristics. Therefore, the production and purification process of nucleic acid drugs does not necessitate specific customization, and it is easy to obtain the natural structure of the desired protein owing to the target cell-endogenous expression mechanisms.^227^

Delivering mRNA into the body for translation into antibodies, rather than directly administering the mAbs, circumvents the hassles associated with protein production and purification. Moreover, this approach enables the encoding of proteins that are challenging to synthesize in vitro. Deal et al.^228^ engineered an mRNA sequence encoding the pathogen-specific immunoglobulin A (IgA) mAbs, which was encapsulated in LNPs and delivered to mucosal secretions. Intriguingly, the pharmacodynamics of lgA_mRNA_ encoded by this mRNA closely resemble those of endogenous human lgA rather than recombinant IgA (IgA_R_). These findings suggested that the mRNA antibody technology held promise for intercepting pathogens on mucosal surfaces, offering a novel and effective approach for the prevention and treatment of diseases.

The intravenous administration of anti-vascular endothelial growth factor (VEGF) antibodies typically results in their predominant distribution in the vasculature and interstitial spaces, with limited accumulation in the target organ—the lungs. This limited localization impairs their therapeutic efficacy for non-small cell lung cancers (NSCLCs) and may have adverse effects on normal tissues. A team led by Le et al.^229^ developed a pulmonary-targeting nanoparticle for delivering mRNA-encoding Bevacizumab, an anti-VEGF antibody widely used in the clinic. These nanoparticles utilized the inherent properties of poly(beta-amino esters) (PBAEs) to achieve their pulmonary-targeting function. This innovative approach effectively suppresses angiogenesis in NSCLCs, offering a promising therapeutic strategy for the treatment of this disease.

The *Orthopoxvirus* genus, including MPXV and variola virus (VARV), constitutes a significant global health threat that causes severe pox diseases in both humans and animals. Chi et al.^230^ constructed mRNA combinations encoding four mAbs (mAbs 22, 283, 26, and 301) specifically designed to target and neutralize vaccinia virus (VACV) A33, VACV B5, MPXV M1 and VACV A27 proteins. This was the first application of mRNA antibodies against Orthopoxvirus in vivo. The results indicated that a single injection of LNPs encapsulated with mRNA in mice could rapidly induce the production of corresponding neutralizing antibodies, which demonstrated significant protective effects in the VACV lethal challenge mouse model and reduced mortality rates.

In the last decade, there have been significant advancements in the treatment of advanced cancers with immune checkpoint inhibitors (ICIs). Currently, clinically approved ICIs consist of innovative medications that target programmed cell death protein-1 (PD-1), programmed death ligand-1 (PD-L1), cytotoxic T- lymphocyte-associated protein 4 (CTLA-4), and lymphocyte activation gene-3 (LAG-3).^231,232^ Nevertheless, ICIs still possess certain limitations, such as the potential for immune-related adverse events.^233,234^ In order to address these safety concerns, researchers have explored the use of mRNA therapies encoding ICIs.^235^ Pruitt et al.^236^ transfected DCs with mRNA encoding the heavy and light chains of anti-CTLA-4 and anti-GITR mAbs, as well as mRNA-encoding tumor antigens. Their findings demonstrated that these mRNA-engineered DC cells significantly augmented antitumor immunity in melanoma-bearing mice without eliciting any signs of autoimmunity. Utilizing this technology, a Phase I clinical trial for patients with metastatic melanoma is presently under assessment (NCT01216436). The IVT mRNA platform also enables the continuous endogenous synthesis of bispecific antibodies that bind to T cells. Zeng et al.^237^ established and optimized an mRNA sequence called Z15-0-2 that codes for a bispecific nanobody of anti-PD-1 and anti-CTLA-4. Moreover, there are mRNA-based therapeutics encoding anti-PD-L1 mAb for cancer treatment^238^ and mRNA-6981 encoding PD-L1 for autoimmune diseases.^239^

Rituximab is a human/murine chimeric glycosylated IgG1-κ mAb with specific affinity for the transmembrane protein CD20 on B lymphocytes.^240^ Thran et al.^241^ developed LNPs formulations containing mRNA-encoding Rituximab (anti-CD20 mAb) to combat B-cell lymphoma. They observed that the mRNA LNPs treatment exhibited a pronounced and potent antitumor effect comparable to that of recombinant antibody therapy.^79^ The mRNA drugs encoding anti-CD20 mAb may also offer promising therapeutic options for autoimmune conditions such as multiple sclerosis and rheumatoid arthritis.^207^ Antibody-based mRNA therapies hold significant potential for the treatment of tumors, chronic inflammation, and autoimmune disorders.^242^

#### Cytokine

Theoretically, mRNA has the potential to encode any protein, including cytokines that can be used as standalone therapies or in combination with other treatment modalities for disease management.^243^

Cytokines play a pivotal role in regulating various biological processes such as cell survival, proliferation, differentiation and immune cell activity by facilitating crucial intercellular communication within short distances.^244^ The potential of cytokines in the realm of antitumor research is exemplified by their capacity to impede the proliferation of tumor cells, facilitate the induction of apoptosis, and modulate both innate and adaptive immunity.^245^ Furthermore, cytokines may exhibit immunosuppressive effects, offering a potential therapeutic approach for conditions derived from hyperactive immune responses such as autoimmune diseases. The cytokine subgroups commonly encompass IL, interferons (IFNs), chemokines, granulocyte-macrophage colony-stimulating factor (GM-CSF) and so on.^244^

##### Immune-potentiating cytokine

**IL-2, IL-21, and IL-15:** The FDA granted approval for the use of recombinant human IL-2 in 1992 for the treatment of metastatic renal cancer, and in 1998 for the management of metastatic melanoma.^246^ However, intravenous administration of IL-2 exhibits limited tumor accumulation^247^ and possesses a short half-life (only 5 to 7 min). In order to achieve enhanced antitumor effects, administration of high doses of IL-2 is imperative, leading to elevated concentrations of IL-2 in the bloodstream and subsequent manifestation of adverse reactions in patients, including asthenia, pyrexia, and potentially life-threatening toxicity such as capillary leak syndrome.^248^ To mitigate the systemic toxicity associated with IL-2 protein delivery, Shin et al.^249^ proposed a novel approach involving intratumoral administration of IL-2 mRNA NPs. These NPs are composed of polyethyleneimine-modified porous silica and effectively minimize off-target translation of mRNA. Jiang et al.^250^ developed mRNA sequences encoding fusion proteins consisting of IL-2, CD25 (IL-2Rα), and a cleavable linker, which were encapsulated within ionizable lipid U-101–derived NPs. The designed linker was susceptible to cleavage by tumor-specific matrix metalloproteinase-14 (MMP-14). Furthermore, the researchers observed that U-101–derived NPs exhibited superior transfection efficacy compared to approved ALC-0315–LNPs, thereby contributing to the platform’s ability to achieve enhanced antitumor efficacy with reduced toxicity. Beck et al.^251^ combined long-lasting mRNA-encoded IL-2 with tumor-targeting mAb therapy, thereby inducing a highly pro-inflammatory TME and overcoming tumor resistance resulting from the absence of MHC I.

In addition to IL-2, other cytokines that belong to the gamma-chain receptor family include IL-4, IL-7, IL-15, IL-21.^252^ The role of IL-21 in the clearance of hepatitis B virus (HBV) is significant. The mRNA LNPs encoding IL-21, delivered by Shen et al.^253^, demonstrated remarkable efficacy in inducing effective clearance of HBV and its covalently closed circular DNA (cccDNA).

IL-15 shares a structural resemblance with IL-2^254^ and plays a crucial role in the regulation of both innate and adaptive immunity.^255^ IL-15 is widely acknowledged as a T-cell growth factor that governs the activation, proliferation, and cytotoxicity of T cells, B cells, and natural killer (NK) cells, thereby enhancing the production of IFN-γ and TNF-α. Moreover, due to its unlikely induction of Treg cell activity during immune stimulation,^254^ IL-15 has been regarded as a promising candidate in oncology.^255^ Nevertheless, IL-15 is associated with significant drawbacks such as severe toxicities and a short half-life. These challenges can be addressed through efficient delivery of mRNA-encoding IL-15 to the tumor. Wang et al.^256^ designed a nanoplatform to specifically target PD-L1 for the delivery of IL-15 mRNA, and integrated it with an ultrasound-targeted microbubble destruction therapy approach. This therapeutic approach not only effectively facilitates the expression of IL-15, but also significantly enhances intracellular reactive oxygen species (ROS) levels, induces immunogenic cell death (ICD), thereby effectively suppressing tumor growth and recurrence. Furthermore, this integrated therapy offers real-time ultrasound imaging guidance. Gavigan et al.^257^ developed a combination therapy comprising IFNα, IL-7, IL-15, and a TNF receptor superfamily agonist. Their findings demonstrated that co-delivery of mRNA mixture encoding IFNα, IL-7, a protein fused with IL-15 and its receptor α chain, and OX40L could elicit potent antitumor immune responses in mice.

**IL-12 and IL-27:** Liu et al.^258^ endeavored to synergize IL-12 mRNA with Oxaliplatin prodrug for the management of colorectal cancer (CRC). Hewitt et al.^259^ discovered in preclinical investigations that intratumoral administration of LNPs loaded with mRNA-encoding IL-12 could augment the antitumor effects by inducing the release of IFN-γ and promoting the transformation of Th1 cells within the TME. The Phase I trial of human IL-12 mRNA (MEDI1191) has been successfully completed (NCT03946800). To further enhance tumor infiltration of IL-12 and mitigate systemic toxicity caused by its extratumoral presence, Trani et al.^260^ engineered mRNAs encoding single-chain IL-12 along with two additional components: a rat anti-mouse AFS98 mAb variable region that targets colony-stimulating factor-1 receptor (CSF1R) and the variable region of Avelumab (anti-PD-L1 antibody), which significantly augment post-transcriptional binding of IL-12 to the tumor.

IL-27, belonging to the IL-12 family, exhibits comparable antitumor effects as IL-12. The activation of T cells and NK cells by IL-12 and IL-27 occurs through distinct mechanisms, whereby IL-12 activates T cells via the signal transducer and activator of transcription (Stat) 4 pathway, while IL-27 engages T cells through both the Stat1 and Stat3 pathways. Liu et al.^261^ developed LNPs loaded with mRNA and observed that co-delivering IL-12 + IL-27 mRNAs using the same LNPs carrier resulted in superior tumor suppression compared to the single delivery of IL-12 mRNA. Furthermore, the incorporation of IL-27, an anti-inflammatory cytokine, could mitigate potential toxic inflammation associated with mono-administration of IL-12. This combination not only enhanced efficacy but also reduced systemic toxicity.

**GM-CSF:** GM-CSF is a cytokine that promotes the generation of various myeloid cell subpopulations, such as DCs, monocytes, macrophages, and neutrophils. Excessive or inadequate levels of GM-CSF could enhance the malignancy of cancer.^262^ The mRNA mixture (SAR441000) encoding IL-12, IFN alpha-2b, GM-CSF, and IL-15 was administered intratumorally as a monotherapy or in combination with Cemiplimab during the Phase I trial (NCT03871348) conducted in 2019 for the treatment of advanced solid tumors. The results demonstrated favorable tolerability and discernible immunomodulatory effects for both SAR441000 monotherapy and its combination with Cemiplimab.^263^

To extend the serum half-life of GM-CSF, it can be conjugated with biologically safe and structurally stable proteins. Yeapuri et al.^264^ developed mRNA LNPs encoding the albumin-GM-CSF fusion protein, thereby prolonging the half-life of the target protein and enhancing drug bioavailability to optimize its therapeutic efficacy.

##### Immune-suppressing cytokine

**IL-2:** Although mRNA-based cytokine therapies are commonly employed in the field of antitumor research, their potential applications in the treatment of other diseases should not be underestimated. The IL-2 receptor exists in two forms with varying affinities, the form expressed by Treg cells exhibiting a higher affinity for IL-2. Consequently, at low concentrations, IL-2 preferentially activates Tregs.^118,119^ However, at higher concentrations, it also triggers the activation of pro-inflammatory T cells and NK cells. Treg cells represent a subset of CD4^+^ T lymphocytes with significant immunosuppressive capabilities.^265^ Impairments in the function of Treg cells or a deficiency in their numbers can result in the breakdown of immune tolerance and the development of autoimmune diseases within the body.^266^ Consequently, induced Treg cells hold promise for the treatment of autoimmune diseases and the restoration of immunological self-tolerance.^267,268^ Picciotto et al.^118^ engineered mRNA to encode a fusion protein of human serum albumin IL-2 mutein (HSA-IL2m) with an extended half-life. Their findings demonstrated that delivering this lipid-encapsulated mRNA could selectively expand Tregs in vivo without activating NK cells or conventional T cells. This approach offers a novel strategy for suppressing autoreactive T cells and restoring immune balance in autoimmune diseases. The mRNA-6231, encoding HSA-IL2m, has already progressed to Phase I clinical trial (NCT04916431).^221^

**IL-4:** IL-4 is involved in the regulation of the activation, proliferation, differentiation, and survival of various T-cell subtypes.^269^ It supports the differentiation of Th2 cells and antagonizes the function of Th1 cells associated with numerous autoimmune disorders.^270^ The modulation of Th-cells subgroups, particularly the balance between Th1 and Th2, exerts a significant influence on the pathogenesis of type 1 diabetes.^271,272^ Creusot et al.^270^ reported a novel therapy that delivered translationally enhanced IL-4 mRNA into DCs using electroporation. They have shown that modified DCs containing mRNA could modulate autoimmune diabetes in nonobese diabetic mice by boosting the functionality of Treg cells and inducing a shift in the proportion of Th cell populations.

**IL-10:** IL-10 is a cytokine with anti-inflammatory properties, capable of suppressing the expression of pro-inflammatory cytokines and inhibiting the presentation of alloantigens by APCs.^273^ Chen et al.^274^ developed a novel delivery system with self-protection and active targeting capabilities for encapsulating mRNA encoding IL-10. Nucleic acids were combined with polyphenols to mitigate enzymatic degradation. Due to the susceptibility of hyaluronic acid to oxidation in the inflamed colon and subsequent inactivation, it is essential to combine bilirubin, known for its potent antioxidant activity, with hyaluronic acid in order to ensure effective targeting of CD44. They demonstrated that rectal administration of the IL-10 mRNA delivery system could effectively induce therapeutic effects in a murine model of colitis. Atherosclerosis is a chronic inflammatory condition. Bu et al.^275^ developed an exosome-based delivery platform for loading engineered IL-10 mRNA. They observed that this platform could effectively mitigate atherosclerosis in ApoE−/− (Apolipoprotein E-deficient) mice.

#### Ligand

The mRNA can also encode immune-stimulating ligands, thereby facilitating an immunogenic response. The TriMix therapy consists of a combination of mRNA encoding three distinct immune-stimulating proteins, namely CD40 ligand (CD40L), CD70, and constitutively active TLR4.^276^ This innovative mRNA-based vaccine can be administered via intratumoral injection for patients with early-stage, resectable breast cancer, which is currently undergoing Phase I clinical trial (NCT03788083).

The TriMix compound not only serves as a direct mRNA vaccine but also functions as an adjuvant in immunization research due to its capacity to encode DC-activating proteins. The ECI-006 vaccine consists of a combination of mRNA-encoding adjuvant proteins (TriMix) and mRNA-encoding melanoma-specific TAAs (tyrosinase, gp100, MAGE-A3, MAGE-C2, and PRAME). A Phase I clinical trial has been conducted to evaluate this mRNA vaccine in patients with stage IIc/III/IV melanoma who have undergone surgical resection (NCT03394937). The results demonstrated favorable tolerability profiles when administering ECI-006 at doses of 600 or 1800 μg to the patients, and immunogenicity was observed in a subset of patients.^277^

#### Tumor suppressor protein

The optimal goal of cancer treatment is to eradicate tumor cells while minimizing the harm to healthy tissues and cells. In pursuit of this objective, suicide gene therapy, a novel approach to cancer treatment, has garnered attention.^278^ Cancer suicide gene therapies can be categorized into three groups: enzyme/prodrug systems, pro-apoptotic genes, and toxins.^279^ The enzyme/prodrug system involves the transfer of genetic material encoding enzymes to tumor cells, leading to the conversion of nontoxic prodrugs by these enzymes into cytotoxic metabolites that induce apoptosis in tumor cells.^278,280^ For instance, the innovative human herpes simplex virus–thymidine kinase (HSV-TK)/ganciclovir suicide gene system shows promise. However, the HSV-TK system is hampered by significant limitations associated with its immunogenicity.^281^ Furthermore, enzyme/prodrug systems also encounter the challenge of off-target effects on normal tissues, resulting in toxicity, as well as suboptimal efficacy against slow-dividing tumor cells. To address these challenges, the approach of developing genes encoding pro-apoptotic proteins and bacterial toxins as suicide genes was employed.^282^

##### Enzyme-encoding mRNA for enzyme/prodrug systems

The combination of cytosine deaminase and uridine phosphoryltransferase (UPRT)/5-fluorocytosine (5-FC) forms an enzyme/prodrug system. The cytosine deaminase is capable of converting the nontoxic 5-FC into the toxic metabolite 5-fluorouracil (5-FU). The gene encoding cytosine deaminase is commonly fused with the UPRT gene to enhance its efficacy.^279^ Mizrak et al.^283^ obtained microvesicles (MVs) loaded with cytosine deaminase-UPRT mRNA/protein by transfecting the cytosine deaminase-UPRT-EGFP plasmid into HEK-293T cells. Subsequently, they observed that intratumoral injection of cytosine deaminase-UPRT-mRNA/protein-containing MVs and treatment with 5-FC significantly suppressed tumor growth in tumor-bearing mice.

Inducible caspase-9 (iC9) is an engineered version of caspase-9 that can be dimerized and activated upon exposure to the chemical inducers of dimerization (CID), such as the AP1903 molecule, subsequently triggering downstream apoptotic pathways and leading to cell death.^284^ Nakashima et al.^285^ developed LNPs carrying iC9 mRNA. They discovered that the combination of iC9 mRNA and CID exhibited cytotoxic effects on three breast cancer cell lines in vitro. On the other hand, Saito et al.^286^ engineered liposomes for the delivery of iC9 mRNA and combined them with CID for the treatment of T-cell malignancies. It was found that the treatment regimen exhibited anti-leukemia therapeutic effects both in vitro and in vivo.

##### Pro-apoptotic protein

**p53 and PTEN**: It is widely acknowledged that cancer originates from the mutation of somatic cells, and that oncogenic mutations or abnormal expression of cellular proto-oncogenes can trigger carcinogenesis. A distinct category of tumor suppressor gene can counteract these carcinogenic effects and impede tumor development. Notably, p53, retinoblastoma (Rb), and phosphatase and tensin homolog deleted on chromosome ten (PTEN) are three pivotal tumor suppressor proteins with interconnected functions.^287^ The majority of human cancers exhibit loss-of-function mutations in the p53 protein. However, due to the fact that p53 is a transcription factor, it has long been deemed undruggable.^288^ Nevertheless, the emergence of mRNA therapies has facilitated the development of numerous p53-based cancer treatments utilizing mRNA.

To augment the therapeutic efficacy of p53 mRNA therapy, it can be synergistically combined with other well-established antitumor modalities. Zhang et al.^289^ developed paclitaxel amino fat-derived NPs for encapsulating p53 mRNA and chemotherapy drugs, demonstrating remarkable antitumor effects in an orthotopic triple-negative breast cancer (TNBC) mouse model. Zhou et al.^290^ were the pioneers in reporting the integration of mRNA therapy with photodynamic therapy (PDT) for tumor treatment. They developed a ROS-responsive NPs platform encapsulating p53 mRNA and indocyanine green (ICG). These NPs can be triggered by ROS to release p53 mRNA, thereby augmenting p53 expression and inducing apoptosis in lung cancer cell. Simultaneously, laser irradiation could activate the released ICG to facilitate PDT. To enhance the expression of intracellular target proteins, Cao et al.^291^ employed a novel strategy by designing the CRISPR/dCasRx-SINEB2 platform to augment the translation of endogenous mRNA-encoding tumor suppressor proteins (including PTEN and p53), thereby inducing suppressed proliferation and increased apoptosis in bladder cancer cells. Liu et al.^292^ established a nanoparticle platform to encapsulate PTEN mRNA for brain delivery. The NPs exhibited enhanced capability to cross the blood-brain barrier and target tumor cells, demonstrating the potential of using mRNA-based technologies for treating brain diseases.

**Bim and Bax:** Bim is a significant pro-apoptotic member within the Bcl-2 protein family.^293^ Gao et al.^294^ developed a delivery system that combined cell-penetrating peptides (CPPs), cRGD-R9, and cationic nano-sized DOTAP-mPEG-PCL scaffolds to deliver mRNA-encoding Bim. The Bim mRNA delivery system exhibited potent inhibitory effects on tumors in various colon cancer models through the induction of mitochondrial apoptosis.

Bax is a pro-apoptotic molecule that can serve as a tumor suppressor protein. Okumura et al.^295^ developed liposomes to deliver mRNA-encoding Bax and compared the antitumor effects with those of a Bax plasmid in tumor-bearing mice. Their findings indicated that the use of Bax mRNA therapy via liposomes exhibited stronger antitumor effects than the Bax plasmid.

**TRAIL:** Tumor necrosis factor-related apoptosis-inducing ligand (TRAIL) can trigger apoptotic cell death in tumor cells while sparing normal cells. Gu et al.^296^ developed mRNA-based LNPs encoding a fusion protein of TRAIL and the receptor-binding domain (RBD) from the SARS-CoV-2 spike protein, which were administered via intratumoral injection in colon cancer mice models. They observed that the RBD-TRAIL mRNA LNPs effectively inhibited tumor growth in mice. Silva et al.^297^ discovered that the direct intratumoral injection of LNPs loaded with TRAIL mRNA could effectively induce tumor cell death, particularly when combined with Losartan or angiotensin 1–7.

**Apoptin:** Apoptin is a protein encoded by the gene of the chicken anemia virus.^298^ It is capable of inducing apoptosis in tumor cells through a p53-independent pathway.^299^ It is noteworthy that Apoptin can trigger apoptosis in tumor cells while sparing normal diploid cells.^298^ Therefore, as a tumor suppressor protein, Apoptin holds promise as a safe anticancer medication.^300^ However, the collection and purification process of this protein is highly intricate and costly.^299^ Therefore, the gene therapy being developed for antitumor Apoptin primarily relies on DNA^301^ or viral vectors.^298^ Tang et al.^299^ engineered nanospheres with self-assembly capabilities to encapsulate mRNA-encoding Apoptin. The combination of Apoptin mRNA with Doxorubicin exhibited remarkable synergistic antitumor effects.

The mRNA-based therapy that encodes tumor suppressor proteins is currently in its early stages of development. Ongoing explorations are being conducted regarding numerous oncogenes and their corresponding tumor suppressor proteins. Qu et al.^302^ proposed that Huntingtin-associated protein-1 (HAP1) may function as a potential tumor suppressor in gastric cancer. Zheng and Song^303^ believed that Synaptopodin 2 (SYNPO2) functions as both a structural protein in muscle tissue and an emerging tumor suppressor protein, supported by its positive correlation with favorable cancer prognosis, thereby indicating its crucial role in cancer prevention and treatment. With the identification of additional tumor suppressor proteins, it is anticipated that mRNA-based therapies encoding these proteins will gradually emerge as a promising avenue in cancer research.

##### Immunotoxin

Immunotoxins are typically chimeric proteins composed of a protein toxin conjugated to an antibody fragment,^304^ enabling specific targeting and elimination of antigen-bearing cells.^305^ The toxic components in immunotoxins typically originate from a variety of sources, including bacteria, plants, and human cells. Bacterial-derived toxins such as diphtheria toxin and pseudomonas exotoxin A (PE), as well as plant-derived toxins like ricin and gelonin, are commonly used. Human-derived toxins include granzymes and RNases. Among these, the application of bacterial and plant toxins is limited due to their potential immunogenicity in humans.^306^ Various strategies have been developed to mitigate the immunogenicity of immunotoxins. These include employing immunosuppressive therapy, utilizing human-derived toxin proteins, and modifying toxin moiety to evade detection by the human immune system.^307^

The short half-life of the immunotoxin in the circulatory system may limit its therapeutic activity. To extend the half-life of immunotoxin in circulation, Guo et al.^308^ fused it with the albumin-binding domain. The production of recombinant immunotoxins is typically conducted in bacteria. However, it is generally challenging to produce soluble proteins with molecular weights exceeding 80 kDa in bacterial systems.^309^ mRNA-based immunotoxin therapy presents a promising alternative.

The application of immunotoxins for the treatment of cancer is currently under investigation, which is, however, constrained by their large molecular weight, limited tumor penetration, and the presence of cellular resistance. Consequently, immunotoxins have not yet received approval for clinical use against solid tumors. To address these challenges, Granot-Matok et al.^310^ developed a LNPs system that delivered modified mRNA encoding the PE domain. This innovative approach employed toxin-encoding mRNA to overcome the limitations associated with immunotoxin therapy, offering improved safety and enhanced therapeutic efficacy.

PE38, a derivative of the potent toxin PE produced by *Pseudomonas aeruginosa*, has been engineered to remove its targeting domain while retaining its toxicity. The toxicity of PE lies in its specific inhibition of elongation factor 2 (EF-2) in the cytosol of eukaryotes. Eggers et al.^311^ utilized PE38 in the development of immunotoxins by fusing it with human VEGF and an anti-human epidermal growth factor receptor-2 (HER2)/neu single-chain variable fragment (scFv). They transfected human primary T cells with mRNA-encoding immunotoxins and demonstrated that the engineered T cells displayed significantly enhanced cytotoxicity against cancer cells in vitro when combined with bispecific antibodies.

The subtilase cytotoxin, diphtheria toxin, and abrin-a belong to the AB toxin family. Their B-subunit initially binds to the target cell, followed by the entry of the A-subunit into the cell, where it exerts toxicity by inhibiting protein synthesis, ultimately leading to the demise of the target cell. Hirschberger et al.^312^ developed three chemically modified mRNAs encoding subtilase cytotoxin, diphtheria toxin, and abrin-a. They demonstrated that the mRNA therapy encoding abrin-a exhibited superior efficacy in reducing the viability of tumor cells in vitro compared to the other two mRNA therapies.

Furthermore, immunotoxins offer a promising therapeutic approach for antiviral treatment. Their mechanism involves targeting and eradicating infected cells, rather than the inhibition of viral or cellular pathways essential for virus replication and spread.^313^ There is great potential for achieving significant advancements in the fields of antitumor and antiviral researches, among others, through the application of immunotoxins.

#### Adoptive cell therapy

Adoptive cell therapy involves the extraction of the patient’s immune cells, followed by ex vivo editing and activation, and subsequent reintroduction into the patient for disease treatment. This approach aims to enhance the identification and elimination of tumor cells. Additionally, there is ongoing development of in situ adoptive cell therapy.^314^ It is noteworthy that the subsequent adoptive cell therapies are founded upon RNA methods, as opposed to conventional DNA methodologies. Specifically, CAR-T therapy should be further subdivided into RNA chimeric antigen receptor T cells (rCAR-T).

##### CAR-T

In the past decade, chimeric antigen receptor (CAR)-expressing T-cell (CAR-T-cell) therapy has made major breakthroughs in the treatment of hematological cancers. Disappointingly, its effectiveness in treating solid tumors is still limited due to a lack of comprehensive understanding of tumor-specific antigens, the suppression of T-cell infiltration and killing ability by the TME, and limited expansion potential of T cells.^315^ Therefore, Mackensen et al. designed CAR-T cells targeting the tumor-specific antigen Claudin 6 (CLDN6) and demonstrated potential clinical activity. However, due to the limited durability of a single dose, they integrated it with a CAR-T-cell Amplifying RNA Vaccine (CARVac) for administration. This combination approach exhibited good tolerability in subjects and showed promise of enhancing CAR-T-cell expansion and improving tumor treatment efficacy. Currently, the study is in Phase I/II clinical trial (NCT04503278).^120,316^ Despite promising results observed in clinical trial, the findings fell short of meeting expectations, underscoring the urgent challenges that still need to be addressed in targeting CLDN6 CAR-T therapy combined with CARVac administration.^315^ The methodology employed in this study for CAR-T-cell production aligns with clinical practice, encompassing ex vivo culturing of CAR-T cells followed by their infusion into patients. This approach entails significant expenditures for manufacturing. Hence, there is an urgent imperative to develop a swift, robust, and economically efficient alternative strategy for manufacturing CAR-T cells. A promising avenue involves the direct delivery of mRNA into the body to induce in situ formation of CAR-T cells.

Despite the challenges associated with transfecting exogenous mRNA into T cells, Tombácz et al.^317^ discovered that the application of CD4 antibody-conjugated mRNA LNPs encoding Cre recombinase or firefly luciferase significantly augmented the signal of mRNA by ~30-fold in spleen T cells and increased the expression of reporter genes in CD4^+^ T cells in vivo when compared to non-targeted mRNA LNPs. Zhou et al.^318^ used CD3 antibody-modified LNPs loaded with IL-6 short hairpin RNA (IL-6 shRNA) and CD19-CAR (based on plasmid DNA) for targeted delivery to CD3^+^ T cells, which resulted in the downregulation of IL-6 within CAR-T cells and a reduction in cytokine release syndrome (CRS), thereby enhancing the safety profile of leukemia treatment. Rurik et al.^319^ developed CD5-targeted LNPs encapsulating mRNA encoding a CAR against fibroblast activation protein (FAP). By administering these CD5-targeted LNPs, potent anti-fibrotic CAR-T cells could be generated in vivo, which mitigated cardiac fibrosis and restored cardiac function.^320^

Given the pivotal role played by autoreactive T and B cells in autoimmune disorders, CAR-T-cell therapy has also become a therapeutic option within the realm of autoimmune diseases.^321^ The pathogenesis of myasthenia gravis (MG) involves the presence of pathogenic plasma cells and autoantibodies, making it a prototypical autoimmune disorder. The ongoing Phase Ib/IIa trial (NCT04146051) involving Descartes-08 rCAR-T cells for the treatment of generalized MG entailed transfecting mRNA-encoding anti-BCMA (B-cell maturation antigen) into CD8^+^ T cells. The findings demonstrated that Descartes-08, which expressed anti-BCMA targeting CAR protein on autologous CD8^+^ T cells, exhibited favorable safety and tolerability profiles in MG patients. Furthermore, sustained reduction in MG severity was observed with continued administration of Descartes-08 infusions over a follow-up period of up to 9 months. These promising outcomes warranted further investigation into the potential therapeutic efficacy of this approach in other autoimmune disorders.^121^ Excitingly, the Descartes-08 anti-BCMA rCAR-T therapy for systemic lupus erythematosus (SLE-001) has successfully progressed into Phase II as of February 2024 (NCT06038474).^322^ On the other hand, Thatte et al.^323^ administered Foxp3 mRNA LNPs to CD4^+^ T cells in order to generate immunosuppressive Foxp3-T cells. These cells have the capacity to suppress the proliferation of effector T cells, highlighting the potential of mRNA-engineered immunosuppressive T cells in the management of autoimmune disorders.

##### TCR-T

In addition to CAR-T-cell therapy, there also exist targeted adoptive cell therapies employing T-cell receptor (TCR) engineered T cell (TCR-T cell). To address hepatocellular carcinoma (HCC) associated with HBV following liver transplantation, Yang et al.^324^ employed electroporation to introduce mRNA-encoding HBV-specific TCR into ex vivo T cells, which were subsequently transferred to the patients. Currently in Phase I clinical trial (NCT02719782), the study demonstrated favorable tolerability of this treatment approach.

Maggadottir et al.^325^ conducted a study on the treatment of metastatic microsatellite instability-high (MSI-H) CRC, wherein they employed an mRNA TCR-modified T-cell therapy approach. This involved administering autologous T cells electroporated with IVT Radium-1 TCR mRNA, specifically targeting the -1A frameshift mutation in the *TGFβRII* gene, to patients with advanced MSI-H CRC. The results demonstrated a favorable safety profile for this TCR-T-cell therapy (NCT03431311).

In addition, also for the treatment of viral-associated tumor diseases, such as HPV-associated cervical cancer, Ling et al.^326^ designed a pH-responsive nonviral nano-carrier to deliver Cas9 mRNA and the guide RNAs (gRNAs) of oncogenes E6 and E7 that target HPV, which can effectively knock out the E6/E7 oncogenes, reverse the tumor’s immunosuppressive environment, and promote CD8^+^ T-cell survival. Therefore, genome editing therapies co-delivering Cas9 mRNA and gRNAs that target oncogenes can also be combined with adoptive T-cell transfer to improve cancer treatment outcomes.

##### CAR-M

The therapeutic efficacy of CAR-T therapy in treating solid tumors is limited, which is greatly derived from the limited infiltration capacity of T cells within the TME. Conversely, macrophages possess the ability to infiltrate solid tumor tissue and engage with almost all cells in the TME, including tumor cells and diverse immune cells. Consequently, exploring the potential of CAR-expressing macrophages (CAR-M) represents a novel avenue for immunotherapy.^327^ The investigational agent CT-0058, a pro-inflammatory macrophage cell product, is currently undergoing Phase I clinical trial for the treatment of solid tumors (NCT04660929). The anti-HER2 CAR expressed in CAR-M cells was obtained via adenoviral vectors rather than mRNA transfection.^328^ Meanwhile, the MCY-M11, currently undergoing clinical Phase I for advanced ovarian cancer and peritoneal mesothelioma treatment, is derived from the transfection of anti-mesothelin CAR (Meso-CAR) mRNA into peripheral blood mononuclear cells (PBMCs) (NCT03608618).^329^

CAR-M therapy not only holds immense potential in combating cancer, but also exhibits promising applications in conducting cutting-edge research on bacterial eradication. Tang et al.^330^ developed LNPs system encapsulating mRNA that encoded a CAR specifically targeting methicillin-resistant *Staphylococcus aureus* (MRSA). The LNPs surface was modified with the CRV peptide (sequence CRVLRSGSC) to facilitate macrophages targeting. This antibacterial CAR-M therapy demonstrated promising potential in eradicating MRSA.

##### mRNA-engineered DCs

DCs are a crucial subset of APCs responsible for presenting TAAs to T cells, and their functionality can be compromised by TME. Although engineered DCs therapy has gained attention, its standalone efficacy remains unsatisfactory, necessitating combination with other therapies to counteract the inhibitory effects of the TME.^331^ The year 2002 witnessed a clinical trial evaluating the efficacy a IVT mRNA DC vaccine against melanoma (NCT01278940).^332^ In 2006, Kyte et al.^333^ reported the results of this Phase I/II trial, indicating that the treatment of tumor-mRNA transfected DCs was safe and feasible, and that the DC vaccine was able to induce antigen-specific T-cell responses in ~50% of patients.

To reprogram the TME and initiate tumor-specific T-cell responses, Zhang et al.^331^ designed two mRNA LNPs. One of these mRNA LNPs encoded CD40 ligand, which elicited potent ICD in tumor tissues, resulting in the expression of TAAs and CD40 ligand on the surface of tumor cells. The other LNPs carried CD40 mRNA with the aim to be internalized by DCs for generating in situ engineered DCs, leading to increased expression of CD40 protein and activation upon interaction with CD40 ligand on tumor cells.

The majority of patients diagnosed with acute myeloid leukemia (AML) exhibit a persistent and recurrent manifestation of the disease attributed to residual leukemic cells, even following standard chemotherapy, resulting in a survival rate of less than 5 years. Consequently, supplementary therapeutic interventions are imperative for eradicating minimal residual disease (MRD). The significant overexpression of Wilms tumor 1 (WT1) in AML and its association with the pathogenesis of the disease render it a promising target for T cells. In light of this, a Phase II clinical trial was conducted in 2010 to evaluate the efficacy of loading autologous DCs with WT1 antigen via mRNA electroporation as an adjunctive treatment strategy for AML patients who had completed chemotherapy (NCT00965224). The findings reported by Anguille et al.^334^ demonstrated that this vaccination approach effectively elicits robust T-cell immune responses in AML patients at high risk of relapse, thereby establishing both the safety and potential utility of autologous *WT1* mRNA-electroporated DC vaccine following chemotherapy.

### Non-immunotherapy

**Fig. 4:**
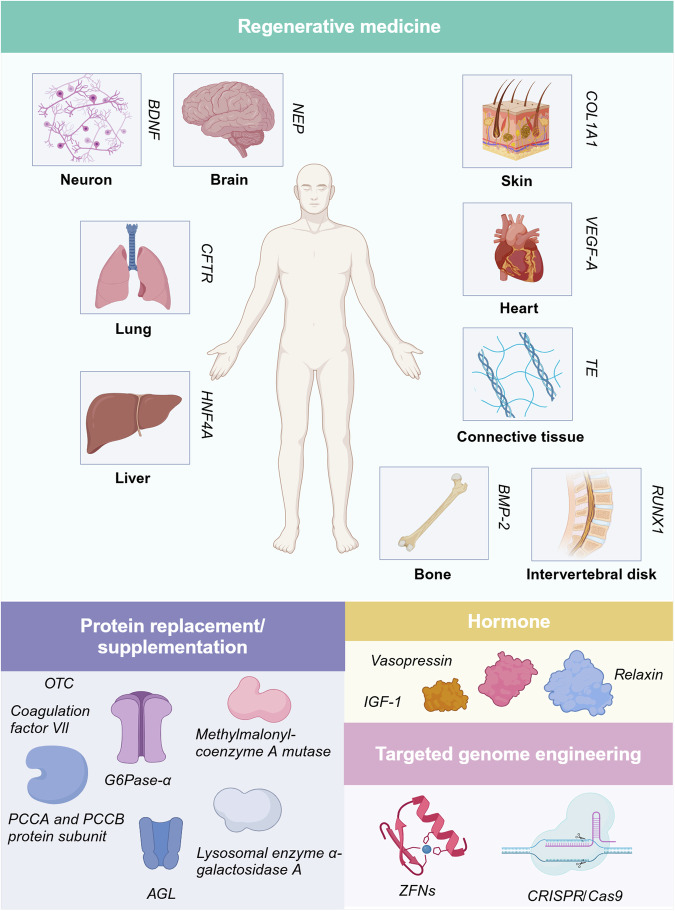
Overview diagram of mRNA-based non-immunotherapy. BDNF brain-derived neurotrophic factor, NEP neprilysin, CFTR cystic fibrosis transmembrane conductance regulator, HNF4A human hepatocyte nuclear factor α, COL1A1 extracellular-matrix α1 type-I collagen, VEGF-A vascular endothelial growth factor-A, TE tropoelastin, BMP-2 bone morphogenetic protein-2, RUNX1 runt-related transcription factor 1, OTC ornithine transcarbamylase, G6Pase-α glucose-6-phosphatase-alpha, AGL amylo-α-1,6-glucosidase 4-alpha-glucanotransferase, IGF-1 insulin-like growth factor 1, ZFNs Zinc finger nucleases, CRISPR/Cas9 clustered regularly interspaced short palindromic repeats/associated protein 9. The graphic is created with BioRender.com

The emergence of mRNA therapies has infused considerable hope into the battle against previously incurable diseases. Theoretically, optimally designed IVT mRNA can be introduced into cells either through in vivo or ex vivo transfection and subsequently translated into biologically active peptide/protein.^335^ The non-immunotherapy described in this article entails directly transcription of mRNA to complement deficient or aberrant proteins within the organism, as well as for gene editing technology, while evading any immune response from the recipient (Fig. 4).

The effective treatment of genetic defect diseases with IVT mRNA relies on multiple factors, encompassing the stability and translational potency of the mRNA, the efficacy and biological activity of the encoded protein, as well as its circulation half-life. Importantly, it is worth noting that the therapeutic threshold necessitates an active protein quantity ranging from milligrams to grams, whereas even at the milligram or nanogram level, antigens can provoke an immune response in the body.^336^ To mitigate the immunogenicity of mRNA therapies, several optimizations can be implemented for IVT mRNA, including refining the nucleotide sequence, employing diverse mRNA nucleoside modifications, optimizing reaction conditions of the IVT conditions to minimize byproducts generation, and purifying the synthesized mRNA.^337^

The repeated administration remains effective in maintaining high protein expression and ensuring in vivo safety, which is crucial for the transformation of mRNA vaccines into mRNA therapeutics. Long-term or even lifelong repeated administration is necessary for treating chronic diseases.^338^ However, delivery vectors, such as LNPs, constitute a crucial component in mRNA-based therapeutics, necessitating careful consideration of their immunogenic potential.

The pattern-recognition receptor family on the cell membrane includes TLR4, which is a crucial member known to be activated by damage- and pathogen-associated molecular patterns (DAMPs and PAMPs). PAMPs encompass lipopolysaccharide (LPS)^339^ and monophosphoryl lipid A (MPLA; a nontoxic TLR4 agonist approved for clinical use).^340^ Kedmi et al.^341^ discovered that the systemic administration of positively charged NPs carrying small interfering RNA (siRNA) could trigger TLR4-dependent immune responses, leading to toxicity. Furthermore, they demonstrated that the inflammatory reaction was primarily caused by cationic components rather than the siRNA. The cationic lipid DiC14-amidine, synthesized by Lonez et al.,^339^ was shown to activate TLR4. Moreover, competition experiments confirmed that the binding site of this cationic lipid to TLR4 differed from that of LPS. Lonez et al.^342^ synthesized a cationic lipid, RPR206252, which effectively triggered the NF-κB pathway and elicited TNF-α, IL-1β, IL-6, and IFN-γ production in human or mouse macrophages. These cascades were reliant on TLR2/CD14 and NOD-like receptor protein 3 (NLRP3) signaling.

Therefore, it is imperative to develop low-immunogenic LNPs with diverse compositions and enhanced organ-selectivity, or explore alternative delivery vehicles to amplify the therapeutic efficacy of mRNA therapies for non-immune diseases.^343^ In addition, the combination of mRNA therapy with immunosuppressants such as Dexamethasone (a steroidal anti-inflammatory drug) not only holds the potential to enhance mRNA transfection efficiency^344^ but also exhibits promise in augmenting the safety and efficacy of non-immunotherapy.

In consideration of the safety of mRNA drugs, it is recommended that preclinical studies encompass the following areas: (1) investigating the correlation between mRNA dosage, carrier properties, and in vivo levels of cytokine secretion as well as complement activation; (2) assessing the potential development of anti-drug antibodies upon repeated administration; (3) evaluating risk of organ damage, particularly focusing on target organs and concerns related to liver toxicity, along with potential accumulation risks associated with carriers.^337^

#### Protein replacement therapy

Protein replacement therapy is a broad term encompassing the supplementation of proteins with functional defects or the substitution of deficient proteins to achieve therapeutic efficacy.^345^ The delivery of therapeutic proteins directly to the target tissue is significantly impeded by factors such as protein size and biochemical properties.^346^ In contrast, mRNA therapies are more likely to bypass these challenges. At present, the use of mRNA translation in vivo to replace the direct delivery of proteins has become a new pillar of protein replacement therapy, and has been extensively studied in various disease areas, including blood diseases (such as Hemophilia A, HemA), and metabolic diseases (such as ornithine transcarbamylase deficiency, OTCD; Fabry disease, FD; Propionic acidaemia, PA).^335^

mRNA therapy provides a promising treatment option for refractory genetic diseases. HemA, a form of hemophilia caused by insufficient expression or gene mutation of coagulation factor VIII (FVIII), results in impaired blood clotting ability and can lead to life-threatening bleeding episodes, including intracranial hemorrhage. Current protein replacement therapies are costly and require frequent administration due to the short half-life of FVIII protein.^347^ In 2023, the FDA approved Roctavian as the first gene therapy drug for severe HemA in adults, utilizing adeno-associated virus vectors; however, concerns arise regarding potential risks such as liver cancer associated with the introduction of DNA sequences from Roctavian products.^348^ mRNA therapy can express the target protein in the cytoplasm without entering the nucleus or inserting the target cell genome, which is much safer than viral vector and DNA therapy.^1^ Chen et al.^347^ designed an LNPs encapsulating mRNA-encoding FVIII protein, which effectively prolonged the duration of FVIII protein expression.

Low levels of key enzyme proteins or lack of enzyme activity resulting from genetic defects can give rise to inherited metabolic disorders (IMDs). Among these, OTCD is caused by the loss of crucial enzymes involved in the urea cycle within the liver, leading to impaired ammonia metabolism. Elevated levels of ammonia in the bloodstream can result in nerve damage and even fatality. Existing approaches, including dietary controls and ammonia scavengers, fail to address the root cause.^349^ Based on this, Prieve et al.^349^ designed a Hybrid mRNA Technology delivery system (HMT) that protected OTC-encoding mRNA against degradation by nucleases while exhibiting hepatocellular-specific targeting capabilities. HMT consists of two types of nanoparticles: di-block polymer micelles and inert LNPs. The polymer comprises three functional domains: (1) GalNAc, which serves as a targeting ligand for liver-specific uptake by binding to the asialoglycoprotein receptor abundantly expressed in liver cells; (2) hydrophilic polymer segments that maintain the solubility of the polymer; (3) polymer segments containing butyl methacrylate (hydrophobic monomer), 2-propylacrylic acid, and 2-(dimethylamino)ethyl methacrylate. This polymeric vesicle was designed to achieve liver-specific targeting and further facilitate the pH-dependent release of mRNA from endosomal organelles to the cytosol. The inert LNPs can shield mRNA from nucleases. This platform is not only safe but also extends the therapeutic effects for OTCD treatment. Currently, ARCT-810 (OTC mRNA LNPs) has entered a Phase II clinical trial (NCT05526066) in OCT patients aged 12 years and older.^350^

FD is an X-linked lysosomal storage disorder (LSD).^351^ Its root cause lies in the pathogenic mutation of the GLA gene (Xq21.3-q22), which leads to a loss of enzyme activity encoding lysosomal enzyme α-galactosidase A. This results in abnormal metabolism of related glycolipids and the subsequent accumulation of glycolipids throughout the body, causing structural damage and functional loss in various tissues and organs, particularly affecting the heart, kidney, and nervous system. The resulting cardiac dysfunction and cerebrovascular events pose life-threatening risks. Although there are clinically approved enzyme replacement therapies (ERTs), they fail to meet medical needs due to their inability to penetrate the blood-brain barrier.^352^ To this end, in 2019, DeRosa et al.^351^ and Zhu et al.^353^, respectively, reported systemically delivery of mRNA-encoding human alpha-galactosidase A (H-α-gal A) using LNPs, both of which indicated that mRNA LNPs was a potential treatment modality for FD.

PA is a life-threatening IMDs caused by a pathogenic mutation in the propionyl-coenzyme A carboxylase α or β (PCCA or PCCB) subunits gene, and there are currently no approved drugs to address this enzyme deficiency. An innovative approach involving two mRNAs encoding normal human PCCA and PCCB protein subunits has entered Phase I/II clinical trials (NCT04159103). Encapsulated within LNPs, these mRNAs are specifically designed for targeted delivery to the liver to restore PCC enzymes. Promising interim results from this trial have demonstrated a favorable safety profile of mRNA-3927 in PA patients. Notably, dose-optimization studies have revealed a remarkable 70% reduction in relative risk associated with metabolic decompensation events (MDEs), highlighting the therapeutic efficacy.^354^

#### Hormone replacement therapy

Hormones produced by various glands in the body serve as crucial signaling molecules that are transported through the bloodstream to target organs, tissues, and cells, playing a pivotal role in maintaining physiological balance. Disruptions in hormonal regulation can give rise to diverse diseases.^355^ During menopause, hormonal fluctuations lead to the manifestation of menopausal symptoms and an increased risk of cardiovascular disorders. To alleviate these symptoms and slow down the progression of cardiovascular diseases, non-protein hormones like estrogen can be administered orally or topically via gel application. Similarly, for conditions such as androgen deficit in aging men (ADAM) or male hypogonadism, supplementation through similar means may also be considered.^356^ However, due to their size and spatial structure, protein hormones cannot be effectively administered orally as they would undergo degradation within the digestive system. Surprisingly, in 1992, Jirikowski et al.^357^ injected mRNA-encoding vasopressin into Brattleboro rats with successful expression of vasopressin in the hypothalamus and showed therapeutic effect (temporary reversal of diabetes insipidus for up to 5 days).

Insulin-like growth factor 1 (IGF-1) is a versatile growth factor primarily synthesized in the liver.^358^ It can regulate the chondrogenesis of mesenchymal stem cells (MSCs) by stimulating their proliferation and facilitating cartilage differentiation. Wu et al.^359^ used modified mRNA (modRNA) encoding IGF-1 to engineer adipose-derived stem cells (ADSCs). The therapeutic effect of engineered ADSCs on Osteoarthritis (OA) is superior to that of natural ADSCs.

Although there have been limited preclinical investigations on mRNA-encoded protein-like hormones, it is exciting that that mRNA-0184 (encoding the Relaxin-2-variable Light Chain Kappa) developed by ModernaTX, Inc. for the treatment of chronic heart failure has entered Phase I clinical trials in 2023 (NCT05659264).

#### Regenerative medicine application

##### Myocardial infarction

In humans, myocardial infarction results in the occlusion of a portion of the heart’s blood supply and the subsequent death of billions of cardiomyocytes, leading to a high mortality rate due to the challenging regeneration of lost cardiomyocytes. This difficulty hinders the repair of damaged heart function. The vascular endothelial growth factor (VEGF-A) has been identified as having angiogenic properties,^360^ making it a potential therapeutic agent for patients with ischemic heart disease. However, its systemic administration is associated with long-term side effects and poses challenges in specifically targeting the heart.^361^ In 2013, Zangi et al.^360^ designed modRNA encoding VEGF-A and found that a single intracardial infusion of VEGF-A modRNA could increase the density of blood vessels around infarction, reduce infarct size and cell death. In addition, compared to mice treated with VEGF-A DNA, those receiving VEGF-A modRNA showed improved survival rates. This improvement can be attributed to careful consideration regarding timing and dosage since prolonged exposure to VEGF-A in the DNA group resulted in increased vascular permeability and cardiac edema. Furthermore, they discussed how mobilization of epicardial progenitor cells played a crucial role in enhancing cardiac function through expansion and differentiation into cardiovascular lineages.

The VEGF-A_165_ mRNA (AZD8601), dissolved in biocompatible citrate saline, has commenced clinical trials. Specifically, the Phase IIa clinical trial (NCT03370887) aims to enhance outcomes in coronary artery bypass graft patients through endocardial injection of AZD8601.^362^ In addition, AZD8601 exhibits promise for treating type 2 diabetes mellitus (T2DM) and is currently undergoing Phase I clinical trials (NCT02935712).^363^ Nawaz et al.^364^ compared VEGF-A protein production in LNPs containing the same amount of VEGF-A mRNA or in extracellular vesicles (EVs) secreted by three different types of cells (cardiac progenitor cells, CPCs; human lung epithelial HTB-177 cells; human umbilical vein endothelial cells, HUVECs) treated with VEGF-A mRNA LNPs. Their findings revealed that when delivered to endothelial cells in vitro, the LNPs carrier produced the highest level of VEGF-A while CPC-EVs demonstrated the lowest protein yield. However, CPC-EVs was the most effective in promoting angiogenesis per given amount of VEGF-A produced. Intriguingly, during intramyocardial injection, EVs-based carriers displayed the highest expression of VEDF-A. Considering that CPC-EVs induce minimal inflammatory cytokine expression compared to other vectors, they are better suited for delivering mRNA to the heart.

The T-box transcription factor 18 (TBX18) plays a pivotal role in the formation and differentiation of the sinoatrial node (SAN).^365^ Wolfson et al.^366^ discovered that direct injection of naked TBX18 mRNA into the myocardium of rats could effectively transfect cardiomyocytes, leading to transient expression of TBX18 and subsequent reprogramming of the cardiomyocytes into pacemaker cells. Furthermore, they observed that compared to adenoviral vectors, mRNA transfection exhibited a significant reduction in off-target gene expression and lower immunogenicity.

##### Liver fibrosis and cirrhosis

In the context of liver diseases, human hepatocyte nuclear factor α (HNF4A) has been recognized as a pivotal regulator of liver cell phenotype, exerting its influence on hepatic stellate cells and liver macrophages by targeting paraoxoase-1. This targeted regulation effectively mitigates liver fibrosis. Yang et al.^367^ designed HNF4A mRNA-loaded LNPs to improve liver fibrosis and cirrhosis in vivo. It was noteworthy that the use of human HNF4A mRNA not only restored the expression of *HNF4A* in mouse liver but also avoided adverse immune reactions, thereby further enhancing its anti-fibrotic efficacy.

##### Cystic fibrosis

The cystic fibrosis transmembrane conductance regulator (CFTR) gene encodes epithelial ion channels responsible for the transport of chloride and bicarbonate, playing a crucial role in maintaining normal physiological functions. Cystic fibrosis is a monogenic disease caused by mutations in the CFTR gene that causes the lungs and other organs to produce thick mucus that blocks the airways of the lungs and makes breathing difficult, leading to lung damage, respiratory failure and chronic lung infections. In addition, cystic fibrosis also affects other organs such as the pancreas resulting in digestive problems and malnutrition. Furthermore, cystic fibrosis can give rise to various complications including diabetes, liver disease, and infertility. Unfortunately, there is currently no cure for this lifelong condition.^276,368^

In 2018, Translate Bio, Inc. conducted a Phase II trial (NCT03375047) involving adults and seniors aged 18 years or older with cystic fibrosis to investigate the safety and therapeutic efficacy of MRT5005—an aerosol containing codon-optimized CFTR mRNA. It was found that the overall safety of patients receiving MRT5005 was good, but fever and allergic reactions occurred. The etiology and mechanism of fever remain unclear, necessitating further research.^369^ Additionally, two more mRNA therapies, namely ARCT-032 (encoding CFTR protein, NCT05712538) and VX-522 mRNA therapy (NCT05668741), have commenced clinical trials in 2023, with the outcomes yet to be disclosed.

##### Degenerative disease

The treatment of bone defects resulting from nonunion, trauma, or craniofacial malformations is challenging and affects millions of individuals. Currently, the delivery of bone morphogenetic protein-2 (BMP-2) shows promise as a treatment approach to address these defects. However, it has been observed that excessive administration of BMP-2 can lead to severe side effects such as significant inflammation and swelling. For heterotopic ossification, local delivery and precise translation of BMP-2 mRNA to produce functional proteins can be achieved. Wang et al.^370^ employed a dual delivery strategy by simultaneously administering BMP-2 mRNA and non-structural protein-1 (NS1) mRNA in a mass ratio of 3:1. Remarkably, their findings demonstrated that this approach induced high expression levels of BMP-2 surpassing previously reported yields. Notably, they are the first to use unmodified mRNA in the field of regenerative medicine since the co-delivered NS1 mRNA encodes an immune evasion protein originally expressed by *influenza A* viruses (A/Texas/36/1991), which effectively inhibits RNA sensors while reducing the production of immune factors to prevent activation of harmful immune responses.

Wang et al.^371^ further explored the delivery platform of BMP-2/NS1 mRNA by incorporating a lipopolyplex (Lip100/His-lPEI/RNA ternary complex, LPR) loaded with BMP-2/NS1 mRNA into a collagen-nanohydroxyapatite scaffolds. The resulting ready-to-use mRNA-activated matrices (RAMs), obtained through freeze–drying, exhibit a remarkable enhancement in the release time of mRNA, extending up to 16 days. This approach enables in situ expression and continuous production of BMP-2 protein, thereby promoting bone formation. Geng et al.^372^ found that compared with the delivery of recombinant protein or single modRNA alone, the co-delivery of human BMP-2 (hBMP-2) and VEGF-A modRNA-modified bone marrow stem cells (BMSCs) increased the expression level of osteogenic-related gene and enhanced the formation of new bone in rats undergoing skull defect surgery. These findings offer a promising therapeutic option for regenerating bone tissue.

Runt-related transcription factor 1 (RUNX1) is a cartilage-anabolic factor, which promotes the proliferation of chondrocytes and increases the expression of cartilage-anabolic markers of chondrocytes. Furthermore, RUNX1 enhances coccygeal disc hydration content while mitigating disc degeneration by minimizing loss of disc cartilage and other constituents. In this regard, Lin et al.^373^ developed RUNX1 mRNA nanomicelles for the treatment of intervertebral disc (IVD) degeneration. Compared to the control group, administration of RUNX1 mRNA nanomicelles significantly maintained a higher disc height in rats with coccygeal disc degeneration and prevented fibrosis of the disc tissue.

Alzheimer’s disease is a degenerative disorder resulting from an imbalance in the metabolic processes of amyloid-beta (Aβ) synthesis and clearance.^374^ Neprilysin (NEP) is known to play an important role in the clearance process of Aβ. Therefore, Lin et al.^375^ designed polyplex nanomicelles based on NEP-encoding mRNA, which significantly reduced the concentration of Aβ in the brain, demonstrating the potential of mRNA-based therapy in brain therapy. In addition, Li et al.^376^ engineered an mRNA encoding brain-derived neurotrophic factor (BDNF) for AD that was packaged on a poly (β amino esters) (PBAE) nanoplatform and delivered directly to the central nervous system. To prevent excessive neuronal activation caused by high translation of BDNF mRNA, they modified the 3’UTR of this mRNA with neuron-specific miRNA-124 target sequences, ensuring degradation by miRNA upon delivery to neurons. By expressing and releasing BDNF in astrocytes to support neurons, the BDNF mRNA effectively enhanced memory function in AD model mice, thus highlighting the potential of mRNA therapy in the treatment of neurological diseases.

##### Connective tissue disorder

Elastin, one of the most enduring proteins (with a half-life of approximately 74 years), is predominantly expressed during the neonatal period. However, with age, elastin synthesis gradually diminishes and eventually ceases in adults. Elastin plays a pivotal role in endowing tissues and organs with elasticity, thereby contributing significantly to the normal functioning of elastic connective tissue. Unfortunately, genetic disorders like Williams–Beuren syndrome (WBS) can lead to mutations in elastin-related genes, resulting in a reduction of over 50% in elastin synthesis. Homozygous elastin null mutants (ELN−/−) succumb shortly after birth. In addition, factors such as aging and sunburn can also lead to elastin reduction. Lescan et al.^377^ synthesized and modified the mRNA-encoding human tropoelastin (TE). Their findings demonstrated that delivery of TE mRNA increased the synthesis of elastin in pig skin by 20%. Furthermore, Golombek et al.^378^ carried out codon optimization and natural nucleotide modification on TE mRNA to improve the translation efficiency and stability of mRNA, thus improving the expression level of TE protein in vivo and in vitro. This study not only provides valuable insights for other conditions necessitating de novo elastin synthesis but also holds promise for diseases like myocardial infarction.

Another key protein in connective tissue is collagen.^379^ You et al.^380^ designed an exosome-based extracellular-matrix α1 type-I collagen (COL1A1) mRNA for anti-aging and treatment of photoaging skin that successfully alleviated UV-induced skin aging through intradermal delivery via microneedle arrays.

#### Targeted genome engineering

Targeted genome engineering is the use of gene editing technology to knock out, insert and replace the target genome sequence to achieve changes in genetic information,^381^ which brings a new way for the treatment of diseases (especially genetic diseases caused by gene defects).^382^ Zinc finger nucleases (ZFNs) and transcriptional activator-like effector nucleases (TALENs) are widely employed in gene editing techniques over a decade ago.^383^ However, their application is complexed and necessitates the design of proteins that specifically bind to target genes.^384^ The advent of clustered regularly interspaced short palindromic repeats (CRISPR)/associated protein 9 (CRISPR/Cas9) systems has significantly propelled the advancement of gene editing technology. CRISPR/Cas9 obviates the need for altering the protein sequences and only requires the design of alternative gRNAs for customization purposes.^385^ There are three typical forms of the CRISPR/Cas9 system: plasmid DNA (pDNA), Cas9 mRNA/sgRNA, and ribonucleoprotein (RNP, Cas9 protein complexed with gRNA). CRISPR/Cas9 pDNA faces the challenge of nuclear localization and has the risks of off-target repeat expression and genome integration; Cas9 RNP delivery has to surmount obstacles related to macromolecular size and instability; while Cas9 mRNA/sgRNA needs to address the instability of single-stranded nucleic acid.^386^

The CRISPR/Cas9 technology can treat diseases by knocking out abnormal genes. The hallmark of wet age-related macular degeneration (wAMD) is vascular dysplasia. At present, anti-VEGF reagents, the first-line drug for the treatment of wAMD, are expensive and require repeated or even lifelong injection. In light of this, Ling et al.^387^ designed a platform that used a lentiviral system to package Cas9 mRNA and VEGF-A-targeting gRNA. The area of choroidal neovascularization (CNV) was reduced by 63% with no detectable off-target effect. Legumain, encoded by the LGMN gene located on human chromosome 14, has been identified in various tumors such as breast and gastric cancers where it is associated with tumor aggressiveness, migratory behavior, and poor prognosis. Wang et al.^388^ used LNPs to co-deliver Cas9 mRNA and LGMN gene-targeting gRNA, and found that this platform held promise as a therapeutic approach to inhibit breast tumor metastasis.

The CRISPR/Cas9 technology can also remove viral DNA from the body. There are 296 million patients with chronic HBV infection in the world, and current clinical treatment can slow the replication of the virus to a certain extent and delay the progression of the disease, but the viral DNA in infected cells cannot be cleared, and HBV DNA integrated into the host body is considered to be a significant source of cancer risk. Moreover, the formation of cccDNA in the liver nucleus and continuous production of progeny virus posed a challenge to the radical treatment of HBV. To address this issue, Yi et al.^389^ used SM-102-based LNPs to deliver Cas9 mRNA and gRNAs together, and employed this CRISPR/Cas9 gene editing technology to achieve targeted site-specific editing. This approach provided an opportunity for continuous elimination of cccDNA and integrated HBV DNA, thereby facilitating a radical treatment strategy against HBV. In addition, to treat HPV-related cervical cancer, Ling et al.^326^ designed a pH-responsive nonviral nanonucleus for co-delivery of Cas9 mRNA and gRNAs targeting HPV oncogenes E6 and E7. This platform allowed for combining Cas9 mRNA/gRNA with adoptive cell therapy to effectively treat cancer diseases.

Although CRISPR/Cas9 technology holds immense potential for the treatment of genetically related diseases and virus clearance, its development has been hindered by the challenges associated with delivering mRNA/gRNA. Currently, CRISPR/Cas9 is predominantly used in the form of Cas9 RNP or pDNA and delivered via viral vectors or electroporation in clinical trials.^386^ However, there are active efforts to develop mRNA/gRNA delivery strategies such as LNPs,^388–390^ lentivirus, and retroviral vectors.^387,391^ We look forward to developing various delivery vectors to advance the clinical application of mRNA/gRNA.

Clinical trials based on Cas9 mRNA/gRNA have been conducted. Transthyretin (TTR) amyloidosis (abbreviated ATTR amyloidosis) is a life-threatening disease caused by the accumulation of misfolded TTR proteins in tissues, particularly in the heart and nerves. NTLA-2001 represents a gene editing therapy based on the CRISPR-Cas9 system that includes LNPs encapsulated with mRNA-encoding Cas9 protein and TTR-targeting gRNA. The interim trial results from Phase I study (NCT04601051) demonstrated that NTLA-2001 effectively reduced serum TTR concentration with only mild adverse events.^392^ Other examples can be found in Tables 2 and 3. Currently, mRNA-based therapies for encoding ZFN, TALEN, and Cas9 are generally in the developmental stage and have the potential to play a crucial role in disease treatment.^393^

**Table 2:** Representative completed and ongoing clinical studies (non-immunotherapy)

Therapy	Disease	mRNA	mRNA-encoded protein	Delivery system	Administration route	Study started	Phase	Status	Sponsor	NCT number
Protein replacement therapy	Ornithine transcarbamylase deficiency (OTCD)	ARCT-810	Ornithine transcarbamylase (OTC)	LNPs	Intravenous infusion	2022-07-06	Phase II	Recruiting	Arcturus Therapeutics, Inc.	NCT05526066
		MRT5201		LNPs	Intravenous administration	2019-12	Phase I/II	Withdrawn	Translate Bio, Inc.	NCT03767270
	Propionic acidaemia (PA)	mRNA-3927	Propionyl-coenzyme A carboxylase α or β (PCCA or PCCB) subunits	LNPs (SM-86, DSPC, cholesterol, and PEG lipid)	Intravenous infusion	2021-04-15	Phase I/II	Recruiting	ModernaTX, Inc.	NCT04159103
	Glycogen storage disease 1a (GSD1a)	mRNA-3745	Glucose-6-phosphatase-alpha (G6Pase-α)	LNPs	Intravenous infusion	2022-06-01	Phase I/II	Recruiting	ModernaTX, Inc.	NCT05095727
	Glycogen storage disease type III	UX053	Amylo-α-1,6-glucosidase 4-alpha-glucanotransferase (AGL)	LNPs	Intravenous infusion	2021-10-18	Phase I/II	Terminated	Ultragenyx Pharmaceutical Inc	NCT04990388
	Methylmalonic acidemia (MMA)	mRNA-3704	Undisclosed	LNPs	Intravenous infusion	2019-05-28	Phase I/II	Withdrawn	ModernaTX, Inc.	NCT03810690
		mRNA-3705	Methylmalonyl-coenzyme A mutase	LNPs (SM-86, DSPC, cholesterol, and PEG lipid)	Intravenous injection	2021-08-06	Phase I/II	Recruiting	ModernaTX, Inc.	NCT04899310
					Injection	2022-03-08	Phase I/II	Recruiting	ModernaTX, Inc.	NCT05295433
Hormone replacement therapy	Chronic heart failure	mRNA-0184	Relaxin-2-variable light chain kappa	Undisclosed	Intravenous infusion	2022-12-05	Phase I	Recruiting	ModernaTX, Inc.	NCT05659264
	Healthy participants			Undisclosed	Intravenous infusion	2024-02-16	Phase I	Recruiting	ModernaTX, Inc.	NCT06243770
Regenerative medicine application	Myocardial infarction	AZD8601	Vascular endothelial growth factor-A (VEGF-A)	None	Epicardial injection	2018-02-05	Phase IIa	Completed	AstraZeneca	NCT03370887
	Type 2 diabetes mellitus (T2DM)			None	Intradermal injection	2016-12-16	Phase I	Completed	AstraZeneca	NCT02935712
	Cystic fibrosis	MRT5005	Cystic fibrosis transmembrane conductance regulator (CFTR) protein	LNPs	Inhalation	2018-05-10	Phase I/II	Unknown status	Translate Bio, Inc.	NCT03375047
		ARCT-032		LNPs	Inhalation	2023-02-15	Phase I	Recruiting	Arcturus Therapeutics, Inc.	NCT05712538
		VX-522 mRNA therapy	Undisclosed	LNPs	Oral inhalation using nebulizer	2023-02-27	Phase I/II	Recruiting	Vertex Pharmaceuticals Incorporated	NCT05668741
Targeted genome engineering	Transthyretin amyloidosis (ATTR) with cardiomyopathy	NTLA-2001	Cas9 protein	LNPs (ionizable lipid, DSPC, cholesterol, PEG2000-DMG)	Intravenous infusion	2023-12-13	Phase III	Recruiting	Intellia Therapeutics	NCT06128629
	Hereditary angioedema (HAE)	NTLA-2002		LNPs	Intravenous injection	2021-12-10	Phase I/II	Active, not recruiting	Intellia Therapeutics	NCT05120830
	Refractory viral keratitis	BD111 CRISPR/Cas9 mRNA	SpCas9	Lentiviral particles	Corneal injection	2020-11-04	Not applicable	Completed	Shanghai BDgene Co., Ltd.	NCT04560790
	HIV	SB-728mR-HSPC	C-C motif chemokine receptor 5 (CCR5) -specific zinc finger nucleases	Autologous hematopoietic stem and progenitor cells (HSPCs)	Infusion	2016-03-10	Phase I	Active, not recruiting	City of Hope Medical Center	NCT02500849
	Transfusion-dependent beta-thalassemia (TDT)	ST-400 investigational product	ZFNs	Autologous hematopoietic stem and progenitor cells (HSPCs)	Infusion	2018-03-29	Phase I/II	Completed	Sangamo Therapeutics	NCT03432364

*LNPs* lipid nanoparticles, *DSPC* distearoylphosphatidylcholine, *PEG* polyethylene glycol, *CRISPR/Cas9* clustered regularly interspaced short palindromic repeats/associated protein 9, *HIV* human immunodeficiency virus, *ZFNs* Zinc finger nucleases

**Table 3:** Representative preclinical studies (non-immunotherapy)

Therapy	Target organ/tissue	Disease	mRNA-encoded protein	Delivery system	Administration route	Ref.
Protein replacement therapy	Blood	Hemophilia A	Coagulation factor VIII	LNPs (ionizable lipid: DSPC: cholesterol: PEG lipid, 50:10:38.5:1.5)	Intravenous injection	^347^
	Liver	Ornithine transcarbamylase deficiency (OTCD)	Ornithine transcarbamylase (OTC)	LNPs (DOTAP, cholesteryl hemisuccinate, cholesterol, PEG lipid); di-block polymer micelle	Intrahepatic delivery	^349^
	Whole body (especially heart, kidneys, and brain)	Fabry disease (FD)	α-galactosidase A	LNPs (C12-200, DOPE, cholesterol, dimyristoyl glycerol-polyethylene glycol analog)	Intravenous injection	^351^
	Whole body (especially heart, kidneys, and brain)	FD	α-galactosidase A	LNPs (ionizable: helper: structural: PEG, 50:10:38.5:1.5)	Intravenous injection	^353^
Hormone replacement therapy	Brain	Diabetes insipidus	Vasopressin	None	Injection into the hypothalamus	^357^
	Bone	Osteoarthritis (OA)	Insulin-like growth factor 1 (IGF-1)	ADSCs	Intraarticular injection	^359^
Regenerative medicine application	Heart	Myocardial infarction	Vascular endothelial growth factor-A (VEGF-A)	None	Intramyocardial injection	^360^
	Liver	Liver fibrosis and cirrhosis	Hepatocyte nuclear factor alpha (HNF4A)	LNPs	Intravenous injection	^367^
	Bone	Bone defects	Bone morphogenetic protein-2 (BMP-2)	Lipopolyplex	Subcutaneously implant	^371^
	Intervertebral disk (IVD)	IVD degeneration	Runt-related transcription factor 1 (RUNX1)	Polyplex nanomicelles (PEG-polyamino acid block copolymers)	Administered into rat coccygeal disks	^373^
	Brain	Alzheimer’s disease	Neprilysin (NEP)	Polyplex nanomicelles (PEG-polyamino acid block catiomer)	Intracerebrovetricular administration	^375^
	Nervous system	Alzheimer’s disease	Brain-derived neurotrophic factor (BDNF)	PBAE polymers	Delivery to the central nervous system by brain ventricle pumping	^376^
	Skin	Photoaged skin	Extracellular-matrix α1 type-I collagen (COL1A1)	EVs	Intradermal delivery (microneedle array)	^380^
	Connective tissue	Williams–Beuren syndrome (WBS)	Tropoelastin (TE)	None	Intradermal injection	^377^
Targeted genome engineering	Eye	Wet age-related macular degeneration	Cas9	Lentiviral system	Subretinal injection	^387^
	Tumor	Breast cancer	Cas9	LNPs	Intravenous injection	^388^
	Viral carcinogenesis	HBV	Cas9	LNPs (SM-102: DSPC: cholesterol: PEG lipid, 50:10:38.5:1.5)	Intravenous injection	^389^

*LNPs* lipid nanoparticles, *DSPC* distearoylphosphatidylcholine, *PEG* polyethylene glycol, *DOTAP* (2,3-dioleoyloxy-propyl)-trimethylammonium chloride, *DOPE* 1,2-dioleyl-sn-glycero-3-phosphoethanolamine, *ADSCs* adipose-derived stem cells, *EVs* extracellular vesicles, *HBV* hepatitis B virus

## The impacts from individual physiological and pathological characteristics

Beginning in late 2019, patients infected with SARS-CoV-2 virus will progress to COVID-19 disease, ranging from asymptomatic infection to life-threatening disease, with particularly high mortality among vulnerable populations (such as the elderly with compromised immunity, individuals with chronic diseases, those use immunosuppressants, and cancer patients).^394^ The U.S. FDA and the European Medicines Agency (EMA) approved mRNA-1273 (Moderna) and BNT162b2 (Pfizer/BioNTech) for eliciting potent B-cell and T-cell immune responses in healthy people to prevent SARS-CoV-2 infection.^395,396^ However, the protective effect of the vaccine wanes over time, which can be observed in healthy subjects and is more pronounced in immunocompromised subjects. Therefore, it becomes imperative to consider administering a third or even fourth dose of the vaccine.

Specifically, Rizzi et al.^394^ recruited patients receiving immunosuppressive therapy for a follow-up trial. They found an absence of protective antibody response after two primary vaccinations, and detectable antibody titers were not produced until a third vaccination. Moreover, given that the induced immune response diminishes gradually after the third vaccination, depending on the patient’s condition, even a fourth immunization was necessary. Nugent et al.^397^ studied the antibody response and pseudovirus neutralization against SARS-CoV-2 wild-type strain, Omicron BA.1, and BA.5 variants in nursing home residents and healthcare workers after primary mRNA vaccination, first and second boosters. They found that antibody titers and neutralizing ability were progressively increasing with each booster, but subsequently diminished over 3–6 months. Considering that individuals with human immunodeficiency virus (HIV) have significantly compromised immunity, many countries have prioritized SARS-CoV-2 mRNA vaccines for this population. It has been observed that after receiving two doses of BNT162b2 vaccine, HIV-infected ones exhibit a more pronounced weakening in the antibody responses than their healthy counterparts, therefore, a third vaccination is recommended. Heftdal et al.^398^ investigated the persistence of cellular and humoral immunity in HIV patients and controls after the third dose of vaccine and found no significant differences in antibody titers and cellular responses between HIV patients and controls at 4 months after the third dose of BNT162b2 vaccine. In conclusion, repeated vaccination is more imperative in susceptible individuals than in their healthy counterparts for the establishment of enduring immune protection.^394^

The above results are consistent with the results of epidemiological studies on hospitalized patients with severe and high-risk COVID-19 (including the elderly and patients with other diseases). However, there still exist significant variations in clinical outcomes among epidemiological patients, suggesting that the underlying cause for symptom disparities between severe COVID-19 patients and asymptomatic carriers may be more profound. Through differential gene screening, Zhang et al.^399^ identified mutations in type-I IFN genes among severe COVID-19 patients, and they found that at least 3.5% of patients with severe COVID-19 had genetic defects in genes associated with TLR3- and regulatory factor 7 (IRF7)-dependent induction and amplification of type-I IFN.

**Fig. 5:**
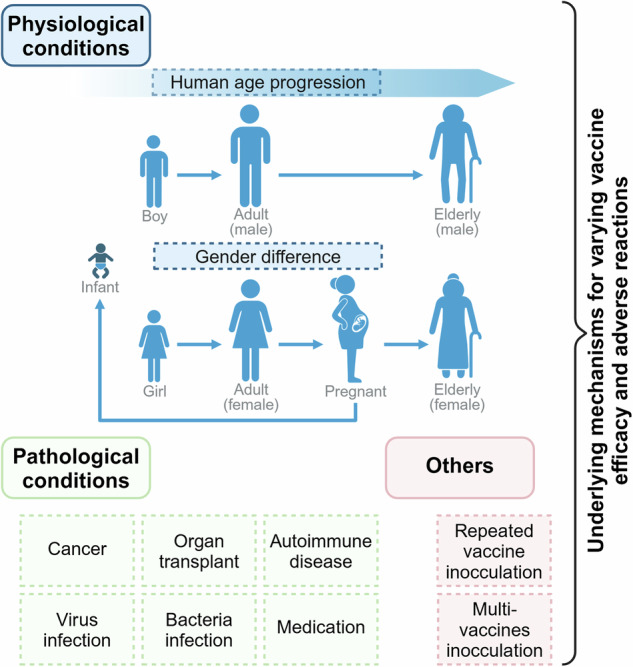
Immunization background map. The variations in individuals’ immunological backgrounds may serve as a potential mechanism for the diversity in vaccine efficacy and adverse reactions following mRNA vaccination. An individual’s immune background is shaped by a multitude of factors, encompassing both physiological conditions and pathological conditions. Moreover, it is crucial to consider the potential adverse effects of repeated or intensive vaccination using the same or multiple vaccines. The graphic is created with BioRender.com

Furthermore, Bastard et al.^400^ found that some individuals who remained severely affected by COVID-19 even after receiving two doses of SARS-CoV-2 mRNA vaccine exhibited not only neutralizing antibodies to the virus but also autoantibodies targeting type-I IFN. The lack of type-I IFN may be the cause of severe hypoxemic pneumonia of COVID-19. The aforementioned evidence implies that it is imperative to focus more on the distinct genetic immune characteristics of vulnerable populations (Fig. 5).

### Age

Vaccination, as a powerful weapon in the prevention and treatment of deadly infectious diseases, can save hundreds of millions of lives by harnessing the human immune response.^401^ However, human immune system is not static, rather, it undergoes profound changes with age.^402^ Age-dependent immune system alterations in the elderly include immune senescence in innate immune responses (i.e., changes in the number, function, and distribution of neutrophils, NK cells, monocytes, and DCs),^403^ and adaptive immune responses (e.g., decreased TCR diversity of aging naïve T cells, shortened telomeres of memory T cells, decreased B-cell pool diversity, and decreased response to foreign antigens).^404^

However, the reasons for decreased vaccine effectiveness in the elderly go far beyond this, that is, it is not only associated with impaired antibody response, decreased T-cell response, and altered antigen presentation of the immune system, but also related to the chronic and systemic aseptic inflammatory state accompanies aging.^405^ Research conducted by Puzianowska-Kuźnicka et al.^406^ has shown that two pro-inflammatory cytokines—IL-6 and C-reactive protein (CRP), increase in the elderly in an age-dependent manner, highlighting the connection between chronic inflammation and aging. Nakaya et al.^407^ conducted studies that revealed an age-related disparity in antibody response following influenza vaccination, irrespective of race or gender, and found that older adults (>65 years) had a relatively reduced B-cell response after vaccination compared to that of younger adults (<65 years). However, these older adults displayed increased monocytes and activated cytotoxic NK cells. Notably, monocytes were also increased in older adults prior to vaccination, suggesting that the baseline state of the immune system in older adults (characterized by age-related low inflammation) is a potential mechanism for the reduced vaccine response.

Age-dependent low-grade chronic inflammation can arise from various potential sources. First, with age, the accumulation of damaged cells and the release of macromolecules such as advanced glycation end-products (AGE) continue to activate the innate immune system and trigger the release of diverse pro-inflammatory factors. Second, immune aging in the elderly may also increase the burden of antigen exposure. For instance, chronic CMV infection not only continuously stimulate the immune system, but also exacerbate both immune aging and inflammation.^408^ Compared to younger individuals, the elderly and the patients with chronic diseases have a pro-inflammatory status and are more prone to triggering cytokine storms following COVID-19 infection, leading to severe COVID-19 clinical symptoms.^408^ Immune aging is an inevitable major risk factor in various chronic diseases, particularly chronic inflammatory conditions including neurodegenerative diseases, cardiovascular diseases, and chronic kidney diseases. In addition, chronic inflammation plays a crucial role in the pathogenesis of various cancers that share a common factor—immune aging. The incidence of these pathological conditions significantly increases with age.^409^ Moreover, immune aging can explain the higher mortality rate observed in older COVID-19 patients due to ineffective T-cell responses and the inability to produce antibodies against SARS-CoV-2. Therefore, identifying markers that reshape immune response in older adults could serve as potential targets for patients with COVID-19 and other age-related ailments.^410^ Immune senescence leads to the disruption of immune checkpoint molecules, such as PD-1 and CTLA-4, and cytokines such as IL-6 and IL-1β.^411,412^ Furthermore, immune senescence is also closely associated with many autoimmune diseases. The incidence of autoimmune diseases appears to rise with age. In turn, autoimmune diseases such as rheumatoid arthritis may also accelerate the process of aging. On one hand, aging induces a state of age-dependent low-grade chronic inflammation, which in turn triggers immune suppression. This exacerbates immune senescence and increases the prevalence of various ailments among older individuals, including chronic inflammatory conditions that further hasten human aging.^409^

Age-related immune senescence and the presence of chronic inflammation in older individuals present significant challenges for disease treatment. Despite the availability of vaccines specifically designed for this population, their overall effectiveness remains inadequate, particularly in infectious diseases such as influenza. Researchers are exploring various therapeutic approaches targeting the pre-vaccination aging characteristics such as inflammation, immune senescence, and mitochondrial dysfunction. Notably, mRNA vaccines have demonstrated promising benefits during the COVID-19 pandemic, including enhanced efficacy among elderly individuals.^413^

During early childhood, the thymus predominantly harbors a substantial population of naive T cells, which subsequently undergo maturation processes. During this period, when encountering pathogens, memory T cells gradually accumulate and overtake the original T cells.^404^ Throughout the process of maturation from infancy to adulthood, a gradual decline is observed in the overall percentage of lymphocytes, as well as in the absolute count of T and B cells. In addition, there is a decrease in the number of NK cells. Interestingly, elderly individuals exhibit significantly higher levels of NK cells, pro-inflammatory cytokines (INF-α, IL-6), and monocyte chemoattractant protein-1 (MCP-1), while demonstrating lower levels of epidermal growth factor (EGF).^414^

The clinical trials of mRNA vaccines are typically conducted across diverse age cohorts, including pediatric, adult, and geriatric populations (Table 4). This may be attributed to variations in their distinct immunological profiles. For instance, mRNA-1273 developed by ModernaTX, Inc. underwent clinical trials in different phases. Initially, it was tested in Phase I (NCT04283461) and Phase III clinical trials (NCT04470427) for individuals aged 18 years and older. Subsequently, it underwent Phase II and III trials for adolescents aged 12 to 18 years (NCT04649151), as well as children aged 6 months to 11 years (NCT04796896). Encouragingly, older adults including participants aged 71 years and older exhibited comparable immune responses to younger adults upon receiving the second dose of the mRNA-1273 vaccine (NCT04283461).^415^ BNT162b2, developed by BioNTech SE, has encompassed age groups and has completed several clinical trials (NCT04368728, NCT04754594, NCT04816643). The findings revealed a decrease in immunogenicity of BNT162b1 or BNT162b2 with advancing age, resulting in a comparatively attenuated overall humoral response among older adults (aged 65–85 years) compared to their younger counterparts (aged 8 to 55 years). Encouragingly, the administration of the second dose of these vaccines could enhance antibody responses in both young and elderly adults (NCT04368728).^416^

**Table 4:** Clinical trials were conducted according to different age groups

mRNA	Disease	mRNA encoded protein	Enrollment (actual)	Ages	Sex/gender	Phase	NCT number	Study started (actual)	Status	Sponsor
mRNA-1273	COVID-19	SARS-CoV-2 spike protein	30415	18 years and older (adult, older adult)	All	Phase III	NCT04470427	2020-07-27	Completed	ModernaTX, Inc.
			4331	12 years to 18 years (child, adult)	All	Phase II/III	NCT04649151	2020-12-09	Active, not recruiting	
			11950	6 months to 11 years (child)	All	Phase II/III	NCT04796896	2021-03-15	Completed	
BNT162b1, BNT162b2	COVID-19	SARS-CoV-2 receptor-binding domain, SARS-CoV-2 spike protein	47079	12 years and older (child, adult, older adult)	All	Phase II/III	NCT04368728	2020-04-29	Completed	BioNTech SE
BNT162b2		SARS-CoV-2 spike protein	16385	12 years and older (child, adult, older adult)	All	Phase III	NCT04955626	2021-07-01	Completed	
			683	18 years and older (adult, older adult, healthy pregnant women)	Female	Phase III	NCT04754594	2021-02-16	Completed	
			11837	6 months to 15 years (child)	All	Phase II/III	NCT04816643	2021-03-24	Completed	
			0	72 days to 102 days (child)	All	Phase I	NCT05630352	2025-01-06 (estimated)	Withdrawn	
mRNA-1345	RSV	RSV prefusion stabilized F (preF) glycoprotein	36557	60 years and older (adult, older adult)	All	Phase II/III	NCT05127434	2021-11-17	Active, not recruiting	ModernaTX, Inc.
			3800	50 years and older (adult, older adult)	All	Phase III	NCT05330975	2022-04-01	Active, not recruiting	
			210 (estimated)	5 months to 24 months (child)	All	Phase I	NCT05743881	2023-02-15	Active, not recruiting	
			1150 (estimated)	18 years and older (adult, older adult)	All	Phase III	NCT06067230	2023-10-06	Recruiting	
			340 (estimated)	2 years to 17 years (child)	All	Phase II	NCT06097299	2023-10-24	Active, not recruiting	
			360 (estimated)	18 years to 40 years (adult, pregnant women and infants)	All	Phase II	NCT06143046	2023-11-15	Recruiting	

*RSV* respiratory syncytial virus

Note: Vaccination safety and immunogenicity are closely related to age, and clinical trials at different ages are needed to determine efficacy

Epidemiological data indicates that individuals aged 60 years and above exhibit a disproportionately higher propensity for severe clinical symptoms and increased rates of hospitalization following SARS-CoV-2 infection during the COVID-19 pandemic. While current vaccines have demonstrated favorable efficacy in younger cohorts, they fail to elicit sustained immunity in the elderly population. Despite previous discussions on potential reasons for this phenomenon, further exploration is warranted to uncover deeper underlying factors. The objective of such investigation is to ultimately develop highly effective vaccines with minimal adverse reactions specifically tailored for older adults. To further enhance the efficacy of the vaccine, in addition to exploring novel strategies against immunosenescence and inflammation, it is also imperative to develop and employ more potent adjuvants.^405^ During the COVID-19 pandemic, mRNA vaccines have accumulated valuable insights into age-related efficacy and adverse reactions of vaccination, thus paving the way for their extensive application in other infectious diseases, tumors, and beyond.

### Gender

Gender dimorphisms are pervasive in human development and physiology. The phenotypic discrepancies of gender derive from a complexed mixture of endogenous and exogenous factors, particularly hormones such as estrogen and androgen. In the era of precision medicine, it becomes crucial to consider the relationship between gender and immune responses for disease prevention and treatment.^417^ The existing literature suggests that male patients demonstrate a higher propensity for developing severe clinical symptoms of SARS-CoV-2 compared to their female counterparts.^418^ This phenomenon may be attributed to the immunomodulatory effects of estrogen, whereas androgen and progesterone exert immunosuppressive actions.^404^ However, the relationship between gender and disease severity is intricate, encompassing the influence of sex hormones, expression of X-linked genes, and other immune regulatory factors. Therefore, a comprehensive understanding of the COVID-19 infection and symptom severity can be achieved by investigating genetic, hormonal, and specific behavioral factors associated with gender and age. Moreover, such researches can contribute to the future development of mRNA vaccines.^418^

Gender plays a pivotal role in determining the risk of HIV infection and its pathogenic progression. Reproductive disparities between genders contribute to an augmented susceptibility of women toward HIV infection. However, during the early stages of HIV infection, women exhibit heightened activation of CD8^+^ T cells and increased expression of interferon-stimulated genes. Nonetheless, post-HIV infection, women face an elevated risk of non-acquired immunodeficiency syndrome (AIDS) morbidity such as cerebrovascular events.^419^

Compared to female mice, male mice demonstrate a higher susceptibility to influenza B virus (FLUBV), mirroring the epidemiological features observed in humans. Cardenas-Garcia et al.^420^ conducted a study encompassing various vaccine designs and found that female mice exhibited more robust lgG and lgA responses and increased CD4^+^ T cells compared to their male counterparts. Furthermore, they reported that both the quality and quantity of immune response were influenced by factors such as vaccine platform, gender, and inclusion of adjuvants, thereby impacting the vaccine efficacy against FLUBV. The findings suggest that, in general, males demonstrate higher morbidity and mortality rates from viral infectious diseases.^421^ However, it is noteworthy that in certain infectious diseases such as measles and dengue, females actually exhibit higher morbidity and mortality rates.^404^

For cardiovascular disease, there are well-established modifiable risk factors such as smoking, obesity, diabetes, hypertension, and hyperlipidemia. In addition to these factors, non-modifiable risk factors such as genetics, age, and gender play a significant role in cardiovascular disease development. The risk of cardiovascular disease significantly increases for males aged 45 years and above, as well as for females who undergo oophorectomy or experience natural menopause.^422^

It is widely acknowledged that sex hormones play a pivotal role in hormone-dependent organ diseases, such as breast and ovarian cancers in females, as well as prostate and testicular cancers in males. The incidence rates of various non-reproductive organ cancers may also exhibit a significant association with gender.^423^ Li et al.^417^ conducted an analysis utilizing data from the UK Biobank and identified 119 diseases exhibiting a gender bias (*P* adjusted < 0.05). Notably, stomach, kidney and lung malignancies demonstrated higher incidence rates in men, while asthma displayed a higher incidence rate in women.

The immune system is intricately interconnected with cancer treatment, and variations in hormonal and X chromosome gene expression can lead to different immune responses among distinct populations of innate and adaptive immune cells.^424^ For example, in patients with KRAS mutations in lung adenocarcinoma, females demonstrate a greater abundance of CD4^+^ T and CD8^+^ T cells compared to males.^425^ The study conducted by Thompson et al.^426^ revealed variations in the functionality of the transcription factor FOXO3, which is associated with tumor-infiltrating DCs tolerance, between genders. These disparities may serve as crucial considerations for augmenting cancer immunotherapy. Observations from clinical trial indicate that immune checkpoint blockade confers greater benefits in the treatment of male cancer patients.^427^ However, certain studies have reported no statistically significant gender-based differences in the efficacy of ICIs.^428^ Additionally, clinical investigations have demonstrated that ICIs amplified therapeutic outcomes in female patients when combined with Cisplatin and/or radiotherapy.^429^ These conflicting experimental findings suggest that the underlying mechanism influencing gender disparities in cancer incidence rates and treatment options are multifaceted and complex.

In summary, women usually exhibit stronger immune responses against infectious diseases and malignant tumors than man. In addition, following vaccination, women demonstrate superior antibody responses, thereby reducing their susceptibility to infections and lowering the mortality rates. However, the better immune response of women also predisposes them to a fourfold increased risk of developing autoimmune diseases.^430,431^ For example, the incidence ratios of multiple sclerosis, scleroderma, and rheumatoid arthritis in females compared to males range from 2:1 to 3:1. However, the gender distribution of systemic lupus erythematosus is remarkably skewed with a ratio of 9:1, representing the most pronounced gender-based disparity among autoimmune diseases.^432^ The etiology of the gender disparity in autoimmune disease incidence is multifaceted and intricate. For instance, the potential exacerbating effect of estrogen on systemic lupus erythematosus, does not preclude its potential immune-protective effect on rheumatoid arthritis.^433^ It is noteworthy that the gender-specific variations in intestinal microbiota may underpin certain gender-related physiological and pathological conditions, thus providing a biological basis for such disparities.^434^

Despite the higher prevalence of autoimmune diseases in women, there is no significant gender disparity in the incidence of immune-related adverse events (IrAEs) among patients receiving ICIs for skin, gastrointestinal, and lacrimal gland-related conditions. However, notable gender differences exist in specific endocrine-related IrAEs. For example, thyroid dysfunction is more commonly seen in women, whereas hypophysitis is more frequently observed in men.^432^

Previously, the role of gender in the development and treatment of diseases was overlooked. For instance, in 1977, the U.S. FDA recommended excluding women of reproductive age from Phase I and early Phase II trials under the guise of safeguarding women and children. Nevertheless, it led to a lack of research and insufficient awareness.^432^ Fortunately, in 2015, the National Institutes of Health (NIH) issued a directive urging researchers to incorporate gender as a biological variable into their research designs, data collection protocols, and outcome analysis.^435^

Currently, there are relatively few mRNA vaccines designed specifically for gender differences. However, the disparities in immune responses resulting from differences in X-linked genes and sex-based hormonal variations do indeed exist, leading to variations in disease susceptibility and clinical manifestations as well as vaccine efficacy. These differences are increasingly being recognized.^436^ Research into the interactions of age and gender may aid in the design of personalized mRNA vaccine treatment strategies tailored to individual patient.^422^

### Pathological condition and vulnerable population

In addition to age and gender, there are other intricate factors affect the efficacy of vaccination. Levin et al.^437^ found that individuals aged over 65 years, males, and groups with immunosuppression-related conditions exhibited significantly lower levels of humoral immunity 6 months after receiving the second dose of BNT162b2. Furer et al.^438^ revealed that patients with autoimmune inflammatory rheumatic disorders displayed a lower humoral response to the two-dose BNT162b2 mRNA vaccine regimen compared to immunocompetent individuals. Although seropositive levels were restored in immunocompetent individuals after receiving the third vaccine dose, only 80.47% of patients experienced restoration of their humoral response. Notably, all patients who received anti-cytokine biologics, Methotrexate monotherapy, Abatacept and Janus kinase inhibitors had their humoral immunity restored. However, only one-third of patients who received Rituximab were able to regain immune protection. The clinical trials regarding vaccine administration in patients receiving immunosuppressants are listed in Table 5.

**Table 5:** Ongoing and completed clinical trials of mRNA vaccines for the treatment of immunosuppressant patients/patients with autoimmune diseases

Background disease	Trial population	mRNA vaccine	Immunosuppressant	Phase	NCT number	Sponsor	Last update posted
Kidney transplant	Recipients who had a kidney transplant at least 6 months ago	Omicron XBB.1.5 vaccine	Everolimus	Phase IV	NCT05924685	University Medical Center Groningen	2024-03-04
Relapsing multiple sclerosis (MS)	Ofatumumab treated participants	Pfizer or Moderna mRNA COVID-19 vaccine	Ofatumumab	Phase IV	NCT04878211	Novartis Pharmaceuticals	2024-02-08
Secondary progressive MS	Patients with secondary progressive MS	COVID-19 modRNA vaccine	Siponimod	Phase IV	NCT04792567	Novartis Pharmaceuticals	2022-12-14
Malignant neoplasms brain	Glioblastoma multiforme patients treated with Temozolomide	Cytomegalovirus (CMV) pp65-LAMP mRNA-loaded dendritic cell (DC) vaccine	Basiliximab	Phase I	NCT00626483	Gary Archer Ph.D.	2021-03-09
Inflammatory bowel disease (IBD)	Patients with IBD	COVID-19 vaccine	Non-systemic immunosuppressive therapy (Mesalamine/Vedolizumab/Vedolizumab combination therapy with Methotrexate or Azathioprine); systemic immunosuppression (Azathioprine/Infliximab/Golimumab/Adalimumab/Certolizumab/Ustekinumab/Tofacitinib/Corticosteroid)	Observational	NCT04818892	University of Wisconsin, Madison	2022-12-07
Glioblastoma (GBM)	GBM patients who underwent resection, Temozolomide (TMZ) therapy, and radiation therapy were also pre-treated with tetanus and given Basiliximab	CMV-specific DC vaccine	Basiliximab	Phase II	NCT02366728	Mustafa Khasraw, MBChB, MD, FRCP, FRACP	2023-06-08
Autoimmune disease	5 autoimmune diseases in adults [systemic lupus erythematosus (SLE)/rheumatoid arthritis (RA)/MS/systemic sclerosis/pemphigus], 4 autoimmune diseases in pediatric participants [systemic lupus erythematosus (SLE)/juvenile idiopathic arthritis (JIA)/pediatric-onset multiple sclerosis (POMS)/juvenile dermatomyositis (JDM)]	COVID-19 vaccine	/	Phase II	NCT05000216	National Institute of Allergy and Infectious Diseases (NIAID)	2024-03-26
Immunosuppress/systematic autoimmunity	Hematological malignancy + immunosuppressant/systematic autoimmunity	COVID-19 vaccine, diphtheria/tetanus toxoids vaccine	e.g., Rituximab, Ocrelizumab, Ofatumumab	Phase III	NCT05415267	Kirby Institute	2024-03-15
Solid organ transplant	Kidney transplant recipients, liver transplant recipients	COVID-19 vaccine	Tacrolimus	Phase II	NCT05077254	NIAID	2024-03-29
MS	MS patients on immunotherapy	COVID-19 vaccine	Ofatumumab, Ocrelizumab, Fingolimod, Siponimod	Observational	NCT05060354	Brigham and Women’s Hospital	2024-01-25
Breast cancer, malignant melanoma	Patients with metastatic breast cancer or malignant melanoma	DC vaccine	Cyclophosphamide (background drugs, as vaccine adjuvants, commonly used as anticancer drugs)	Phase I	NCT00978913	Inge Marie Svane	2015-08-19
Immunocompromised, immunosuppressed	Patients with immunocompromising conditions in the United States	COVID-19 vaccine (BNT162b2)	/	Observational	NCT05020145	Pfizer	2024-02-12
Brain stem glioma	Brain stem glioma patients treated with radiotherapy and Temozolomide	DC vaccine	/	Phase I	NCT03396575	University of Florida	2023-11-18

Note: Follow all entries found under “Search for: mRNA vaccine, immunosuppressant |Card Results| ClinicalTrials.gov”

Leston et al.^439^ classified immunosuppression and correlated it with the mortality rate of COVID-19. Their findings revealed that patients with solid organ transplants and malignant tumors exhibited a significantly higher mortality risk following COVID-19 infection compared to that of immunocompetent individuals. Furthermore, in comparison to immunocompetent individuals, patients with rheumatological conditions and HIV demonstrated a slightly elevated mortality risk. The study established a close association between specific subgroups of immunosuppression and the mortality risk associated with COVID-19 infection. This categorization method holds substantial reference value for targeted treatment strategies and focused vaccination prevention. Meeraus et al.^440^ discovered that individuals with comorbidities and vulnerable populations exhibited reduced vaccine effectiveness following administration of AZD1222 (ChAdOx1 nCov-19) in comparison to those with normal immune systems, while immunosuppressed individuals demonstrated the lowest vaccine effectiveness after the initial dose of AZD1222. Obeid et al.^441^ conducted a comparative analysis on the persistence of humoral responses against SARS-CoV-2 and its variants of concern (VOCs) in immunocompromised patients who received two doses of BNT162b2 or mRNA-1273 vaccines, as well as in healthy individuals. The findings revealed that approximately 50% of patients with solid tumors and hematological malignancies, about 70% of autoimmune disease patients and solid organ transplant recipients, and 40% of healthy individuals had lost protective nAbs 6 months after vaccination. Moreover, it was observed that the durability of binding IgG anti-spike antibodies against SARS-CoV-2 was four to nine times greater than that of nAbs. Furthermore, variations were noted in the immune efficacy between different mRNA vaccines, with BNT162b2 inducing lower magnitude and shorter duration of nAbs compared to mRNA-1273. Therefore, it is imperative to select appropriate mRNA vaccines for diverse vulnerable populations in order to ensure effective administration of vaccinations.

In addition to COVID-19 virus infection, similar scenarios seem to arise in other infectious viruses as well. A study conducted by Mbonde et al.^442^ revealed that immunosuppressed individuals with neuroinvasive West Nile virus (NWNV) exhibited more severe clinical manifestations compared to immunocompetent populations, thereby indicating a worse prognosis and higher risk of adverse reactions. The survival rate of sepsis survivors is significantly diminished following secondary infection, primarily due to the prolonged state of immunosuppression that ensues after acute infection. A comprehensive study conducted by Liao et al.^443^ revealed a close association between IL-10 production by Siglec-F^+^ neutrophils and the suppression of T-lymphocyte activity in this phenomenon. Furthermore, their findings indicated that depletion of neutrophils could enhance T-lymphocyte proliferation and improve T-lymphocyte activity, thereby ameliorating the survival rate among immunosuppressed mice subjected to secondary infection. However, further investigation is required to assess the systemic effects associated with neutrophils depletion.

Persistent immune suppression is observed in people with HIV, which is closely associated with low CD4 counts or abnormal CD4/CD8 ratios. The prevention and treatment of cancer in HIV-infected individuals face challenges due to disparities in health status.^444^ Evidences suggested that individuals infected with HIV might undergo premature aging. The research conducted by Gianesin et al.^445^ further demonstrated that this phenomenon extended to HIV-infected children, resulting in accelerated biological and immunological senescence, particularly affecting the CD8^+^ cell subpopulation. Vergori et al.^446^ discovered that the administration of a third dose of the COVID-19 mRNA vaccine to individuals with HIV could elicit a robust immune response. However, the magnitude of the SARS-CoV-2-specific T-cell response in HIV-positive subjects was comparatively lower than that observed in the HIV-negative control group. In addition, they provided quantitative analysis regarding the potential augmentation of immune levels through extra booster doses for individuals with HIV.

Pregnant women and infants are also considered vulnerable populations. Antibodies derived from COVID-19 vaccines can offer protection against severe virus infections in newborns through the transfer of placental antibodies. There is limited data on the antibody levels of infants, particularly preterm infants, following maternal vaccination. Therefore, Kachikis et al.^447^ conducted a study which revealed that there was no disparity in maternally derived SARS-CoV-2 anti-spike IgG levels between full-term and preterm infants. However, these levels were closely associated with the concentration of the maternal anti-spike antibodies. In addition, it was observed that receiving two or fewer doses of the COVID-19 vaccine might not provide optimal immune protection for pregnant women, nor for infants via cord blood. Consequently, careful consideration should be given to the timing of vaccination for pregnant women.

### Adverse reaction

Johnston et al.^448^ discovered an adverse reaction manifested as a delayed hypersensitivity at the injection site subsequent to administration of the Moderna COVID-19 vaccine. Cappelletti-Montano et al.^449^ discovered that the majority of severe adverse reactions reported after COVID-19 vaccination were associated with cardiac complications, particularly among male adolescents. In addition, thrombosis and dyspnea emerged as prevalent serious symptoms following COVID-19 vaccination. In contrast, the adverse reactions after vaccination with HPV and influenza seemed to be more diverse. Dizziness, loss of consciousness, dyspnea, and convulsions occurred after HPV vaccination, and Guillain-Barré syndrome occurred after influenza vaccination. Vaccination with COVID-19 vaccine (BNT162b2 mRNA or mRNA-1273) can also be associated with the development of various autoimmune diseases, such as autoimmune hepatitis or nephritis, rheumatoid arthritis, and new-onset systemic lupus erythematosus. Heil^450^ endeavored to investigate the underlying factors contributing to a diverse range of life-threatening complications in patients infected with SARS-CoV-2. Although the COVID-19 pandemic seems to be receding, the lessons learned from the development of COVID-19 mRNA vaccines are expected to have implications for non-COVID-19 diseases.

The study conducted by Pettini et al.^451^ demonstrated that multiple administration of mRNA-1273 vaccine effectively enhanced the specific memory B-cell and antibody responses against SARS-CoV-2 in allogeneic hematopoietic cell transplantation patients. Furthermore, they emphasized the significance of repeated vaccination. The phenomenon of extensive and consecutive immunization has become apparent owing to repetitive vaccinations intended to augment vaccine efficacy, along with advancements in vaccines designed for diverse diseases. Nonetheless, this intricacy presents difficulties in evaluating vaccine safety accurately. Interactions between vaccines have the potential to produce favorable or detrimental outcomes alike. Mawson and Croft^452^ postulated that multiple vaccinations might induce activation of the retinoid cascade, resulting in a sequence of unfavorable reactions. The uncertainties of the risks associated with vaccination necessitate further research to optimize vaccine safety.

The safety and immunogenicity profiles following vaccination were assessed in clinical trials through the administration of two distinct vaccine formulations in a predetermined sequence. For example, the Phase I trials of the investigational vaccines BNT164a1 and BNT164b1 have recently commenced with volunteers who have previously received the BCG vaccine (NCT05547464).^453^ Several clinical trials are currently underway to assess the safety and immunogenicity of co-administering two vaccines simultaneously (Table 6). Moreover, mRNA vaccines can be engineered to encode several epitopes from multiple pathogens simultaneously.^454^ Currently, there is ongoing development of multi-component vaccines aimed at achieving progressively more comprehensive disease prevention (Table 7).

**Table 6:** Concurrently administered vaccines

Vaccine	Disease	Study started (actual)	Phase	Status	Sponsor	NCT number
9vHPV + mRNA-1273	Papillomavirus infection and COVID-19	2022-03-28	Phase III	Completed	Merck Sharp & Dohme LLC	NCT05119855
mRNA-1345 + influenza vaccine	RSV and influenza	2023-09-25	Phase III	Active, not recruiting	ModernaTX, Inc.	NCT06060457
V110/V114 + mRNA-1273	Pneumococcal infection and COVID-19	2022-01-12	Phase III	Completed	Merck Sharp & Dohme LLC	NCT05158140
ARCT-2303 + influenza vaccine	COVID-19 and influenza	2024-03 (estimated)	Phase III	Not yet recruiting	Arcturus Therapeutics, Inc.	NCT06279871
BNT162b2 (Omi XBB.1.5) + RIV	COVID-19 and influenza	2024-01-31	Phase II	Active, not recruiting	Pfizer	NCT06237049

*HPV* human papillomavirus, *RSV* respiratory syncytial virus

**Table 7:** Multi-component vaccines

Vaccine	Disease	Study started (actual)	Phase	Status	Sponsor	NCT number
mRNA-1045	Influenza and RSV	2022-10-14	Phase I	Completed	ModernaTX, Inc.	NCT05585632
mRNA-1230	Influenza, RSV and COVID-19	2022-10-14	Phase II	Completed	ModernaTX, Inc.	NCT05585632
mRNA-1083	Influenza and COVID-19	2023-04-14	Phase I/II	Active, not recruiting	ModernaTX, Inc.	NCT05827926
mRNA-1073	Influenza and COVID-19	2022-05-13	Phase I/II	Completed	ModernaTX, Inc.	NCT05375838

*RSV* respiratory syncytial virus

## Conclusions and prospects

### Challenges in mRNA drug application

Despite substantial advancements in the field, effectively and safely delivering mRNA therapeutics to the target site remains a significant challenge. The half-life of direct intravenous administration of mRNA is very short due to their susceptibility to degradation by nucleases. The negative charge and high molecular weight of mRNA hinder cellular transcytosis across the biological membrane. Additional barriers include high renal clearance, non-specific tissue distribution, and off-target effects. In addition, mRNA that enter cells face the challenge of escaping from endosomes.^455^ Furthermore, exogenously introduced mRNA can be recognized by the TLRs and cytosolic nucleic acid receptors, triggering innate immune responses and accelerating the deactivation of IVT mRNA within the body.^181^

Although the application of mRNA delivery systems, LNPs in particular, mitigates certain of the above obstacles, it is important to remain cautious of the potential hazards associated with delivery vehicles, such as inflammation, immunogenicity, and cytotoxicity.^105^ In addition to the toxicity caused by the delivery system, it has been discovered that the production methods, routes of administration, and even proteins generated by the complexed mRNA drugs present toxicity concerns.^456^

### Main reasons for failed mRNA application

Following intravenous or intramuscular administration, mRNA LNPs are extensively distributed in the liver and spleen, potentially resulting in pathological alterations within these organs, such as the disruption of hepatic cell regulation of fatty acid metabolism. In addition, mRNA nanomedicines may elicit adverse immune reactions, such as systemic complement activation, hypersensitivity responses, and cytokine-mediated effects post-vaccination.^98,456^ The adverse immunogenicity caused by LNPs, besides the lipid components, is closely associated with their size, charge, and aggregation characteristics. Smaller LNPs are more advantageous in mitigating the activation of undesired immune responses. On the other hand, it is important to investigate the translation accuracy of nucleotide-modified mRNA therapeutics, as unintended protein synthesis may result in unforeseen outcomes.^457^ Mulroney et al.^458^ discovered that the use of m^1^Ψ-modified IVT mRNA led to a notable increase in the +1 ribosomal frameshift during translation. Understanding the translation process of nucleotide-modified mRNA is essential for future mRNA design and optimization to mitigate potential reductions in efficacy and increases in toxicity.

The success application of mRNA therapies is intricately linked to the complexity and public understanding of targeted disease. Take the extensively studied cancer mRNA vaccine as an example. To date, there is few Phase III mRNA vaccine available for tumors, which may be due to the following reasons: (1) The mRNA encoding a single tumor antigen may inadvertently induce mutations in tumors to evade immune response, necessitating the need for developing mRNA vaccines capable of simultaneously targeting multiple TAAs or TSAs; (2) The surface antigens are distinct across different tumor types, calling for personalized tumor-mRNA vaccines for individual patient. However, the production and implementation of personalized vaccines are often associated with high costs and pose challenges to their widespread clinical application; (3) Tumors exert immunosuppressive effects by impeding the recognition of neoantigens and suppressing the viability of antitumor immune cells, emphasizing the importance of meticulous selection and application of adjuvants for potentiated immune responses; (4) The efficacy of mRNA vaccines is largely limited by its instability, inefficient delivery, and poor transfection efficiency. In these cases, iterative optimization of the mRNA molecule and delivery vehicle is needed; (5) Tumor patients often exhibit compromised immune responses due to a multitude of factors, including advanced age, concurrent medication (e.g., chemotherapy), and comorbidity (e.g., HIV). Hence, in addition to bolstering the immune response with adjuvants, vigilance against potential adverse reactions should be taken into serious consideration; (6) The formidable challenge of eradicating advanced tumors using mRNA vaccines alone necessitates the integration of multiple therapies for synergistic therapeutic effects, such as adoptive cell therapy.^459^

### Efforts and improvements made to address these challenges

The current landscape of mRNA vaccines with promising clinical prospects often comprises three components: the mRNA sequence encoding target protein, delivery vector, and adjuvant.^460^ In certain cases, the mRNA carrier itself functions as vaccine adjuvant.^460^ Notably, although the increased immunogenicity of mRNA drugs boosts immune response, it hampers mRNA translation and suppresses target protein production. In this consideration, the technology of immune-silencing IVT mRNA by replacing naturally occurring nucleosides into the mRNA sequence has been developed.^461^ Employing modified mRNA represents a strategic approach to both enhance protein expression and mitigate aberrant immunogenicity.^462^ In addition, by optimizing the mRNA sequence, such as introducing multiple adenines at the Poly (A) tail, mRNA stability can be further enhanced with the protein translation efficiency improved. Moreover, purification methods including cellulose and fast protein liquid chromatography can be employed to eliminate the immunogenic double-stranded RNA generated during mRNA manufacturing, thereby reducing the inflammatory responses unfavorable to mRNA stability.^463^

In the context of immunotherapy, to avoid the insufficient activation of APCs, especially DCs, with nucleoside-modified immune-silenced mRNA following vaccination,^115^ adjuvants can be incorporated into the delivery system. An ideal adjuvant can lower the dose of antigen-encoding mRNA per vaccine dose, reduce the frequency of vaccination, enhance the robustness and specificity of immune responses, and in certain cases, assume the role of a carrier for the efficient delivery of mRNA and contribute to the stabilization of the vaccine formulation.^464,465^ Gu et al.^466^ used TLR2/6 agonist Pam2Cys (a simple synthetic metabolizable lipoamino acid) as an adjuvant to facilitate mRNA encoding CT26 neoantigen peptide or SARS-CoV-2 spike protein antigen for induction of effective immune responses against cancer or infectious diseases. On the other hand, the adjuvant can be incorporated directly into the mRNA sequence. Li et al.^395^ developed a multiply adjuvanted mRNA vaccine by fusing antigen-encoding mRNA with C3d (the terminal degradation product of mammalian complement component C3)-based mRNA adjuvant, which evoked a protective immune response against SARS-CoV-2 at a dosage ten times lower. Of note, an increasing number of studies have revealed that adjuvant not only enhances the magnitude of immune response to vaccines, but guide the type of adaptive immune response.^467^ Zhu et al.^468^ fabricated mRNA LNPs and investigated the differential immune responses elicited by mRNA LNPs supplemented with the natural STING agonist 2'3’-cyclic GMP-AMP (cGAMP) and/or the mucosal adjuvant alpha-galactosylceramide (αGC). The results indicated that mRNA LNPs containing cGAMP alone induced a stronger cellular immune response than mRNA LNPs containing both cGAMP and αGC, which was consistent with higher levels of IgG and IgG2a antibody responses, as well as better protection against homologous viral challenge. The study also demonstrated that mRNA LNPs containing cGAMP could accumulate CD4^+^ T cells in the lung, while mRNA LNPs containing αGC could enlarge the iNKT cells in the spleen. Activated iNKT cells may exhibit potent cytotoxicity, indicating the paramount importance of the judicious selection and application of adjuvants. At the cellular level, it is well-established that Actinidia eriantha polysaccharide (AEPS) elicits a mixed CD4^+^ Th1 and CD4^+^ Th2 immune response, while Alum induces a strict Th2 response. Nevertheless, many intricate molecular mechanisms remain unclear. Du et al.^469^ conducted a comparative analysis of the innate immune responses elicited by the two adjuvants, and found that AEPS induced higher mRNA expression levels of C-X-C motif chemokine ligand (CCL) 2, CCL3, CCL4, CXCL10, IL-12β, IL-23α in immune effector process, and rapidly recruited neutrophils and monocytes. In comparison, Alum induced IL-7A, IL-17F, and IL-17RA, and recruited neutrophils and eosinophils.

The delivery and storage challenges of mRNA can be resolved through optimization of the delivery vehicle.^115^ To date, a plethora of delivery systems for mRNA have been developed. Among them, LNPs has progressed in clinical practice most rapidly. LNPs not only possesses the capacity to encapsulate and transport mRNA, but also shields the mRNA from nuclease degradation. However, the use of LNPs still presents certain issues, such as the potential damage to cell membranes and organelles due to the cationic characteristics of the ionizable lipid. The development of biodegradable ionizable lipids appears to offer some solutions to this problem.^102,105^ It is crucial to recognize that no ionizable lipid is universally optimal for all nucleic acid formats, and variations in nucleic acid modification, size, and structure can impact the effectiveness of LNPs delivery.^470^ On the other hand, the off-target accumulation of mRNA drugs may lead to suboptimal outcomes. In this case, various stimulus-responsive nanotechnologies have been developed to utilize specific internal stimulus within the cellular microenvironment, including the expression of specific enzymes, increased levels of ROS, hypoxic environments, and changes in the pH or temperature, as well as external stimulus such as light irradiation, magnetic fields, and ultrasound. These stimuli-responsive nanoplatforms have the potential to enhance tissue targeting, promote exosome escape of mRNA, improve the therapeutic effects of mRNA therapy, and reduce toxicity and side effects.^471^ The active targeting of mRNA drugs to desired biological sites through LNPs surface modification is also widely used. Several preclinical studies have already demonstrated the successful delivery of mRNA LNPs to target tissues and cells.^472–474^ For instance, Kim et al.^475^ utilized LNPs modified with tumor-targeting peptides to encapsulate mRNA-encoding PETN. This PETN mRNA LNPs specifically induces ICD in mice 4T1 breast tumor, thereby eliciting effective antitumor immune responses. Tang et al.^476^ engineered LNPs with sialic acid for DCs targeting and efficient endosomal escape. Furthermore, it is anticipated that the next-generation LNPs with the ability to target drugs specifically to distinct intracellular organelles, such as mitochondria and the nucleus, will serve as an innovative paradigm.^472^ In addition to the optimization strategies mentioned above, the delivery system for mRNA can be optimized to enhance the precision of mRNA-based therapies for diseases by adjusting factors such as drug dissolution, diffusion rate, pharmacokinetics, toxicity, and half-life.^477^

### Potential of mRNA drugs to treat various diseases

The mRNA vaccine has emerged as a forefront line in disease prevention and treatment.^276^ The rapid development and regulatory approval of mRNA vaccines against SARS-CoV-2 during the COVID-19 pandemic have demonstrated the advantages of mRNA technology,^478^ exerting a profound influence on its application in infectious diseases and cancer immunotherapy. The Phase III clinical trial of SARS-CoV-2 mRNA vaccine is currently the most clinically available. Additionally, a series of mRNA vaccines against other infectious diseases are under development. For example, the Phase I study (NCT04528719) of mRNA-1345 encoding the prefusion stabilized F (preF) glycoprotein of RSV demonstrates favorable tolerability profiles in both young and elderly individuals, providing the foundation for further investigations where high-risk adults and solid organ transplant patients are enrolled (Phase III, NCT06067230).^479^ Meanwhile, compared to mRNA-1345, the development of mRNA-1010 for seasonal influenza is progressing at a faster pace, which has successfully completed a Phase III trial with 22,510 adults aged over 50 years (NCT05566639). mRNA-based immunotherapies have also been extensively researched for antibacterial and anti-parasite applications. Of note, aggressive fungal infections actually result in a higher annual mortality than malaria, yet there are very few mRNA-based treatments available for fungal infections. We aim to increase awareness of the potential of mRNA-based therapies for treating fungal diseases.^480^

In the context of antitumor immunotherapy, increasing mRNA vaccines has entered the clinic. For example, mRNA-4157 encodes up to 34 patient-specific tumor neoantigens is used in combination with Pembrolizumab for the treatment of melanoma, which has entered Phase IIb clinical trial (NCT03897881).^481^ Presently, the primary focus of research lies in the identification and design of mRNA sequences that target specific cancer antigens for the purpose of achieving personalized cancer mRNA vaccine therapy. Furthermore, while the majority of existing cancer mRNA vaccines are designed for therapeutic purposes rather than preventive measures, there is also significant potential for prophylactic cancer mRNA vaccines.^181^ Given the design flexibility and adaptability of mRNA, the development of personalized cancer mRNA vaccines to optimize antitumor effects based on diverse tumor types and individual patient variations represents a crucial avenue for future advancement.^482^

It should be mentioned that mRNA-based immunotherapy not only has the potential to enhance immune responses against a wide range of infectious diseases and cancers, but also offers the possibility of modulating the immune response for the treatment of autoimmune conditions. mRNA therapeutics designed for autoimmune diseases can be tailored to encode disease-specific antigens or immunosuppressive cytokines. For instance, the mRNA-6231 encoding HSA-IL2m, intended for the treatment of autoimmune disorders, has entered Phase I clinical trial (NCT04916431).^221^

In contrast to immunotherapy where the incorporation of adjuvants is imperative, it is of vital importance for non-immunotherapy to mitigate the immunogenicity of mRNA drugs and to amplify the expression of encoded target proteins.^483^ mRNA-based therapeutics function to replenish deficient proteins or substitute abnormal proteins that exhibit diminished functionality/activity. For example, mRNA therapies for OTCD (ARCT-810, Phase II, NCT05526066; MRT5201, Phase I/II, NCT03767270), PA (mRNA-3927, Phase I/II, NCT04159103), MMA (mRNA-3705, Phase I/II, NCT05295433), and myocardial infarction (AZD8601, Phase IIa, NCT03370887) have entered clinical trials (Table 2).

In this review, we summarize the preclinical and clinical progresses of mRNA drugs in both immunotherapy and non-immunotherapy, and highlight the importance of focusing on host-specific variations for maximized efficacy and safety. Although in the early stages of development, the therapeutic use of mRNA has demonstrated great potential in combating multiple diseases.^484^ The mRNA drugs described in this review primarily encompass traditional IVT mRNA. However, as mRNA technology constantly innovating and advancing, other forms of mRNA drugs, such as saRNA, trans-amplifying mRNA (taRNA), and circRNA, hold immense applicability. It is worth noting that successful clinical implementation of these next-generation mRNA drugs requires the involvement of suitable delivery vehicles and further modifications to the mRNA molecule itself for better tolerability and efficacy.^485^
